# Farnesylated prelamin A induces fibroblast polarity defects in premature aging disorders by inhibiting nesprin-2–SUN2 LINC complex function

**DOI:** 10.1242/jcs.264488

**Published:** 2026-05-18

**Authors:** Chonkit Lio, Yuexia Wang, Paige C. Wilson, Yutao Li, Susumu Antoku, Cecilia Östlund, Ji-Yeon Shin, Howard J. Worman, Wakam Chang, Gregg G. Gundersen

**Affiliations:** ^1^ Faculty of Health Science, University of Macau, Taipa, Macau, China; ^2^ MOE Frontier Science Centre for Precision Oncology, University of Macau, Taipa, Macau, China; ^3^ Department of Medicine, Columbia University, New York, NY 10032, USA; ^4^ Department of Pathology and Cell Biology, Columbia University, New York, NY 10032, USA; ^5^ Department of Biological Sciences, Columbia University, New York, NY 10027, USA

**Keywords:** Cell polarity, Centrosome orientation, Nuclear movement, Prelamin A, ZMPSTE24, Progeria

## Abstract

Disrupted processing of prelamin A (encoded by *LMNA*) causes Hutchinson–Gilford progeria syndrome (HGPS) and related premature aging disorders. The farnesylated prelamin A variant produced in HGPS, termed progerin, alters actin–nuclear interactions mediated by nesprin-2 and SUN2 linker of nucleoskeleton and cytoskeleton (LINC) complexes, resulting in defective cell polarization. To explore further how prelamin A causes these cellular defects, we examined other disease-causing variants that prevent cleavage of lamin A or reduce the activity of the processing enzyme ZMPSTE24. Accumulation of prelamin A or an uncleaved variant in cells reduced diffusional mobilities of nesprin-2 and SUN2 and inhibited their function in cell polarization in a farnesylation-dependent manner. Expression of short carboxyl-terminal tail fragments of prelamin A variants disrupted cell polarity in a farnesylation-dependent fashion. These results show that retention of the farnesyl moiety in the tails of prelamin A or its variants is the common element responsible for disrupting actin force transmission to the nucleus in premature aging syndromes and support the idea that altered function of actin-dependent LINC complexes is a critical component of premature aging.

## Teaser

**Summary:** Farnesylated carboxyl tails of prelamin A variants associated with premature aging disorders disrupt mobility of LINC complex proteins and their roles in nuclear positioning and cell polarization.

## INTRODUCTION

The nuclear lamina is composed of intermediate filament proteins called lamins that polymerize to form meshworks on the inner aspect of the inner nuclear membrane. The lamin A/C gene (*LMNA* in humans, *Lmna* in mice) encodes lamin A and lamin C, which are expressed in most terminally differentiated somatic cells (Lin and Worman, 1993). Lamin A is derived from its precursor prelamin A, which persists only transiently (Gerace et al., 1984; Laliberté et al., 1984; Lehner et al., 1986). The carboxyl terminus of prelamin A ends in a cysteine–aliphatic–aliphatic–any amino acid (CAAX) motif (Fisher et al., 1986). This motif triggers a series of post-translational modifications. Protein farnesyltransferase first catalyzes the addition of an isoprenoid farnesyl moiety to the cysteine (Reiss et al., 1990; Schaber et al., 1990). Subsequently, Ras converting CAAX endopeptidase 1 (RCE1) or zinc metallopeptidase STE24 homolog (ZMPSTE24) cleaves the peptide bond between the cysteine and -AAX (Kim et al., 1999; Leung et al., 2001; Otto et al., 1999; Schmidt et al., 1998). In a third step, isoprenylcysteine carboxyl methyltransferase (ICMT) methylates the carboxyl-terminal farnesylcysteine (Clarke et al., 1988; Dai et al., 1998). Farnesylated and carboxymethylated prelamin A then undergoes a final reaction to generate mature lamin A: an endoproteolytic cleavage removes the carboxyl-terminal 15 amino acids, including the farnesylcysteine (Beck et al., 1990; Weber et al., 1989). The carboxyl-terminal cysteine must be farnesylated for this cleavage reaction to occur (Beck et al., 1990; Sinensky et al., 1994). Results from knockout mice and *in vitro* biochemistry show that ZMPSTE24 is the sole enzyme responsible for this endoproteolytic cleavage (Pendás et al., 2002; Bergo et al., 2002; Corrigan et al., 2005).

Processing defects that lead to the persistence of farnesylated prelamin A variants lead to premature aging (progeroid) syndromes in humans and mice. Hutchinson–Gilford progeria syndrome (HGPS) is caused by a *de novo* G608G (or G608S) mutation in *LMNA* that creates an aberrant splice donor site leading to an in-frame deletion of 50 amino acids from prelamin A, including the ZMPSTE24 cleavage site (De Sandre-Giovannoli et al., 2003; Eriksson et al., 2003). After -AAX cleavage, this internally truncated variant, termed progerin, has the same last six amino acids of prelamin A, including the farnesylcysteine.

Deletion of *Zmpste24* in mice leads to accumulation of full-length farnesylated prelamin A and progeroid phenotypes (Bergo et al., 2002; Pendás et al., 2002). In humans, variants of *ZMPSTE24* cause progeroid disorders (PDs), the severity of which ranges from relatively mild, such as mandibuloacral dysplasia type B (MDB), to neonatal lethal, such as restrictive dermopathy; the severity correlates with residual proteolytic activity of the enzyme (Agarwal et al., 2003; Barrowman et al., 2012; Moulson et al., 2005; Navarro et al., 2005; Shackleton et al., 2005). Previously, we identified a patient with a PD, whose cells accumulated farnesylated prelamin A as a result of a heterozygous *LMNA* L647R variant, which abolishes the ZMPSTE24 recognition site (Wang et al., 2016).

Blocking farnesylation with a farnesyltransferase inhibitor (FTI) reverses abnormal nuclear architecture in cells expressing prelamin A or farnesylated variants (Capell et al., 2005; Glynn and Glover, 2005; Mallampalli et al., 2005; Toth et al., 2005; Wang et al., 2010, 2016; Yang et al., 2005). FTI treatment also significantly improves the phenotypes of *Zmpste24* knockout mice and mice expressing progerin (Capell et al., 2008; Fong et al., 2006; Yang et al., 2006). Hence, it appears that the farnesyl moiety on these persistently expressed proteins contributes to pathology. Based on these results, an FTI has been studied in clinical trials for children with HGPS and ‘processing-deficient progeroid laminopathies’ such as MDB, and has been approved by the US Food and Drug Administration (Dhillon, 2021; Gordon et al., 2018).

Although farnesylation of prelamin A and progerin clearly contributes to their pathological effects, the retention of additional amino acid residues in the tails of these proteins might also have consequences. This is suggested by the milder phenotypes in the patient whose cells express non-cleavable full-length prelamin A L647R (Wang et al., 2016). Abnormal phenotypes of *Zmpste24*^−/−^ mice can be eliminated by deleting one *Lmna* allele, suggesting that the severity of disease depends on levels of farnesylated prelamin A (Fong et al., 2004).

It is unclear how accumulation of prelamin A affects nuclear function. The altered nuclear shape of cells expressing progerin and prelamin A (Capell et al., 2005; Glynn and Glover, 2005; Mallampalli et al., 2005; Toth et al., 2005; Wang et al., 2010; Yang et al., 2005) suggests that factors controlling the structure and mechanics of the nucleus are affected. In fact, the expression of progerin and uncleavable prelamin A variants stiffens nuclei, which might contribute to their altered shape. Progerin also affects the linker of nucleoskeleton and cytoskeleton (LINC) complex (Arsenovic et al., 2016; Chang et al., 2019), which mediates transduction of mechanical forces to and from the nucleus (Lele et al., 2018; Luxton et al., 2010; Maurer and Lammerding, 2019). The LINC complex is composed of nesprins (KASH domain-containing proteins) in the outer nuclear membrane and SUN proteins in the inner nuclear membrane, where they associate with the lamina (Chang et al., 2015; Crisp et al., 2006; Starr and Fridolfsson, 2010). Of the two widely expressed SUN proteins (SUN1 and SUN2), SUN1 protein levels are elevated in progerin-expressing cells (Chang et al., 2019; Chen et al., 2012). SUN1 might also bind with higher affinity to prelamin A (Chen et al., 2014; Crisp et al., 2006; Haque et al., 2006).

SUN2 function appears to be the most affected in nuclei expressing progerin. Fluorescence recovery after photobleaching (FRAP) measurements of the mobility of LINC complex proteins in progerin-expressing cells showed reduced mobility of an actin-binding chimera of nesprin-2G (mini-nesprin-2G or mini-N2G), SUN2 and emerin, but not the mobility of SUN1 or nesprin-3 (Chang et al., 2019). SUN2 supports nesprin-2G coupling of the nucleus to actin filaments but not to microtubules (Luxton et al., 2010; Zhu et al., 2017). Consistent with this, progerin expression blocks actin-dependent nuclear movement in fibroblasts (Chang et al., 2019) and reduces actin-dependent tension on nuclei, as measured by a nesprin-2-based tension probe (Arsenovic et al., 2016). Whereas progerin expression inhibits nuclear actin coupling, it enhances interaction of the nucleus with microtubules (Chang et al., 2019). This presumably reflects the ability of progerin to increase nuclear SUN1, which supports microtubule coupling (Zhu et al., 2017). Blocking SUN1 or the microtubule motor dynein in progerin-expressing cells restores actin coupling of nuclei, indicating that it is the excessive nuclear microtubule coupling that inhibits nuclear actin coupling (Chang et al., 2019). Notably, the SUN domain of SUN1 is responsible for promoting association of nesprin-2G with microtubules and mediating its inhibitory effect on actin-dependent nuclear movement (Li et al., 2026).

**Fig. 1.**
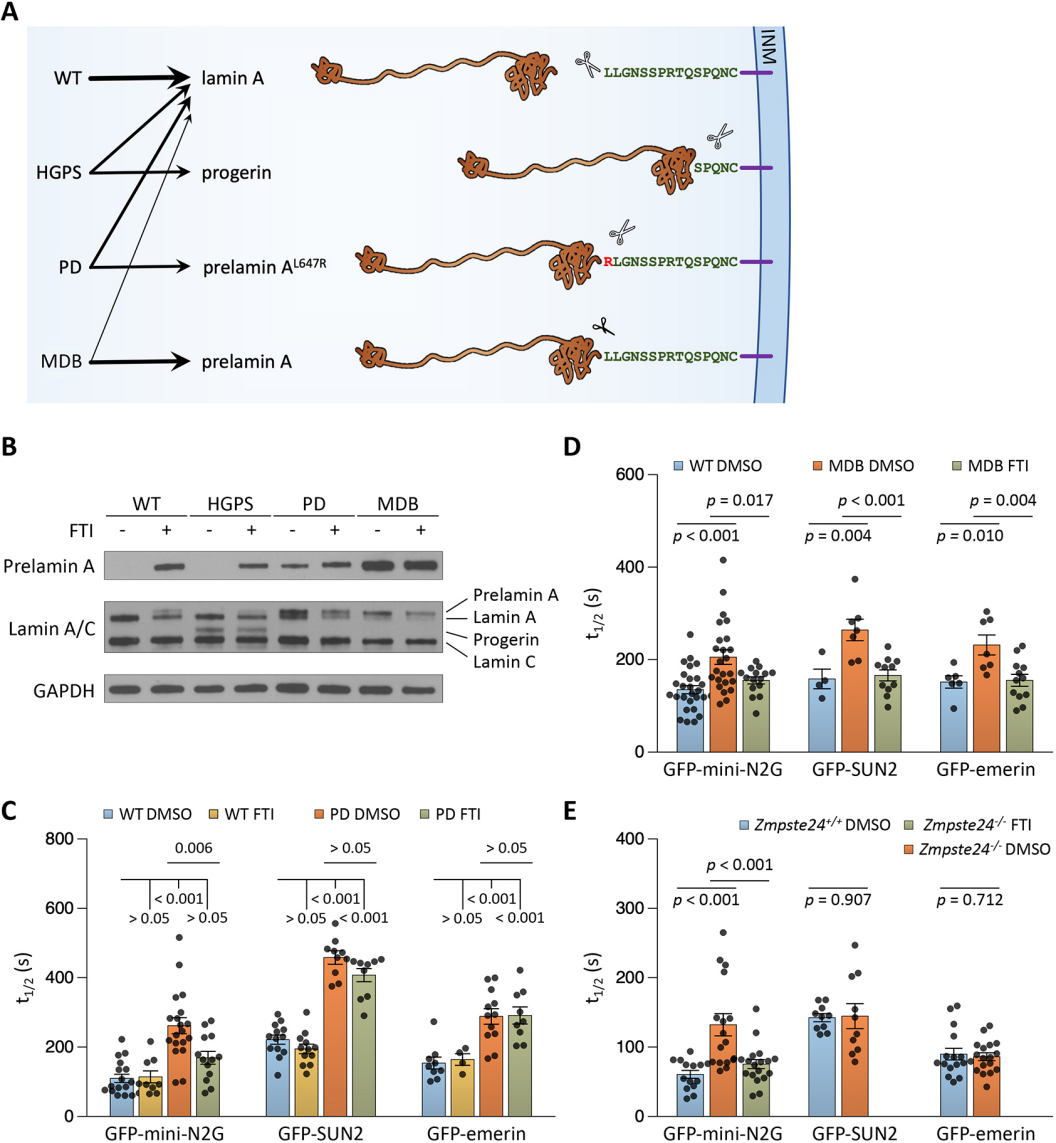
**Expression of prelamin A and its variants differentially affect the diffusional mobilities of mini-N2G, SUN2 and emerin in the nuclear envelope of human and mouse fibroblasts.** (A) Diagrams showing the carboxyl-terminus of prelamin A and its variants. In wild-type (WT) fibroblasts, the carboxyl-terminus of prelamin A, including the farnesylcysteine, is rapidly processed by ZMPSTE24 (depicted as scissors) and only the processed mature form of lamin A is present. In Hutchinson–Gilford progeria syndrome (HGPS) and progeroid disorder (PD) fibroblasts, half of the encoded lamin A lacks either a 50 amino acid segment including the ZMPSTE24 proteolytic site (HGPS) or has a single amino acid substation that abolishes this site (PD). In mandibuloacral dysplasia type B (MDB) fibroblasts with ZMPSTE24 deficiency, some of the farnesylated prelamin A is not cleaved. INM, inner nuclear membrane. (B) Representative immunoblots of protein extracts from the indicated fibroblasts treated with DMSO or the farnesyltransferase inhibitor (FTI) FTI-277 for 72 h. Blots were probed with antibodies to prelamin A, lamin A/C recognizing prelamin A, lamin A, progerin and lamin C, and GAPDH as a loading control. Blots represent seven independent experiments. (C–E) Half times (*t*_1/2_) of fluorescence recovery after photobleaching (FRAP) for the indicated EGFP fusions in the nuclear envelope of WT and PD fibroblasts (C), WT and MDB fibroblasts (D), and *Zmpste24*^+/+^ and *Zmpste24*^−/−^ mouse embryonic fibroblasts (MEFs) (E). Cells were pretreated with either DMSO or 2.5 µM FTI-277 for 24 h. Values are means±s.e.m. from *N*=3 biological replicates. Each dot represents an individual measurement from a single cell. *P*-values (indicated in the graphs) were calculated by one-way ANOVA with post hoc Tukey's test or unpaired two-tailed Student's *t*-test.

Studies with FTIs support the idea that one element of the cellular toxicity of progerin is through its retained farnesyl moiety. Much less studied and understood is whether the retention or loss of different amino acid residues that result from the disrupted processing of progerin and prelamin A (see Fig. 1A) contribute to their toxicity. Here, we tested the role of altered carboxyl-terminal residues and farnesylation in different prelamin A variants and progerin to decipher their relative mechanistic contributions to altering the behaviors and functions of the LINC complex. Our results strongly suggest that the retained farnesyl moiety plays a dominant role in disrupting actin-dependent LINC complex function and that the extra amino acid residues in the various forms of incompletely processed prelamin A do not contribute significantly.

## RESULTS

### Lamin A and prelamin A levels in human fibroblasts expressing prelamin A or progerin variants

In humans, *ZMPSTE24* loss-of-function variants lead to the accumulation of farnesylated prelamin A and the *LMNA* L647R variant leads to a variant differing from prelamin A by a single residue (L647R). Both these proteins differ from mature lamin A by retaining the last 15 amino acids and the farnesyl moiety of prelamin A. After -AAX cleavage, progerin has the same last six carboxyl-terminal amino acid residues, including the farnesylated cysteine, but lacks the preceding 50 residues due to an in-frame deletion resulting from altered RNA splicing (Fig. 1A). We used two antibodies, one that detects lamin A, lamin C, progerin and prelamin A, and one that specifically recognizes prelamin A, to characterize the levels of the proteins in extracts of dermal fibroblasts from patients with *LMNA* and *ZMPSTE24* variants. In wild-type (WT) fibroblasts, lamin A and lamin C were present, but prelamin A was undetectable (Fig. 1B). In fibroblasts from a boy with HGPS, lamin A, progerin and lamin C were detected, but prelamin A was undetectable (Fig. 1B). Fibroblasts from a 17-year-old woman with PD caused by the heterozygous *LMNA* L647R point variant contained prelamin A, lamin A and lamin C, with lamin C being the most abundant (Fig. 1B). In fibroblasts from a 5.8-year-old boy with MDB caused by a homozygous *ZMPSTE24* L425P variant, which both renders the ZMPSTE24 enzyme less active and decreases its stability (Spear et al., 2018), the level of prelamin A was higher than in the PD fibroblasts and a trace amount of lamin A was detected (Fig. 1B). The MDB fibroblasts showed similar levels of lamin C as the PD fibroblasts. Quantification revealed significantly more prelamin A in MDB fibroblasts compared to that in PD fibroblasts (Fig. S1). Prelamin A expression in the WT and HGPS fibroblasts was increased when lamin A maturation was inhibited using a farnesylation inhibitor (Fig. 1B; Fig. S1).

### Diffusional mobilities of nesprin-2G, SUN2 and emerin in fibroblasts expressing farnesylated prelamin A

Progerin expression in both NIH3T3 and HGPS fibroblasts selectively alters the diffusional mobilities of three nuclear envelope proteins, namely, SUN2, nesprin-2G and emerin, in a farnesylation-dependent manner. The diffusional mobility of SUN1, however, is not affected (Chang et al., 2019). We tested whether the expression of prelamin A or variants that retain their farnesyl moiety had a similar effect on the diffusional mobilities of these proteins. In fibroblasts expressing green fluorescent protein (EGFP) fusions of these proteins, we measured FRAP to determine diffusional mobilities. Because of the large size of nesprin-2G, we used EGFP–mini-N2G, a chimeric variant that binds actin and functionally supports actin-dependent nuclear movement (Luxton et al., 2010). The diffusional mobilities of all three nuclear envelope proteins were significantly reduced in PD fibroblasts compared with those in WT fibroblasts (Fig. 1C; Fig. S2). The mobility of EGFP–mini-N2G, but not that of EGFP–SUN2 or EGFP–emerin, was restored in the PD fibroblasts when treated with an FTI, and the lack of effect of FTI treatment in WT cells confirmed that it restores the diffusional mobilities by blocking farnesylation of prelamin A (Fig. 1C; Fig. S2). Similarly, the diffusional mobilities of all three proteins were significantly decreased in MDB fibroblasts but became similar to those in WT fibroblasts after FTI treatment (Fig. 1D; Fig. S3).

We also examined diffusional mobilities of the three proteins in *Zmpste24*^−/−^ mouse embryonic fibroblasts (MEFs) that express only farnesylated prelamin A and no lamin A (Bergo et al., 2002; Fong et al., 2004). The diffusional mobility of EGFP–mini-N2G was reduced in *Zmpste24*^−/−^ MEFs and FTI treatment restored it to a value near that in *Zmpste24*^+/+^ fibroblasts (Fig. 1E; Fig. S4). Diffusional mobilities of EGFP–SUN2 and EGFP–emerin were similar in the fibroblasts from *Zmpste24*^−/−^ and *Zmpste24*^+/+^ mice (Fig. 1E; Fig. S4). Thus, expression of farnesylated prelamin A, similar to expression of progerin, consistently reduces the mobility of nesprin-2G in a farnesylation-dependent manner. Diffusional mobilities of SUN2 and emerin are reduced by farnesylated prelamin A expression but only in human fibroblasts.

### Polarization of migratory human fibroblasts expressing farnesylated prelamin A

The diffusional mobilities of nesprin-2G, SUN2 and emerin were affected in fibroblasts that accumulate farnesylated prelamin A or its variants. These proteins function together in nuclear movement and centrosome orientation during cell polarization for migration (Chang et al., 2013; Luxton et al., 2010). Both processes are impaired in fibroblasts from children with HGPS but can be restored by inhibiting protein farnesylation (Chang et al., 2019). We assessed nuclear movement and centrosome orientation in human fibroblasts that accumulate prelamin A using a wounded monolayer assay in which the serum factor lysophosphatidic acid (LPA) activates Cdc42-dependent pathways that trigger actin and myosin II-dependent rearward nuclear movement away from the leading edge, while microtubules maintain centrosome position at the cell center (Gomes et al., 2005; Palazzo et al., 2001; Schmoranzer et al., 2009). Dorsal actin cables couple to and move the nucleus by engaging nesprin-2G and SUN2 LINC complexes that assemble into transmembrane actin-associated nuclear (TAN) lines (Gomes et al., 2005; Luxton et al., 2010). Cytoplasmically oriented emerin on the outer nuclear membrane and perinuclear myosin-IIB maintain the rearward direction of actin cable flow and consequential nuclear movement (Chang et al., 2013). The result is reorientation of the centrosome to a position between the nucleus and the leading edge.

**Fig. 2.**
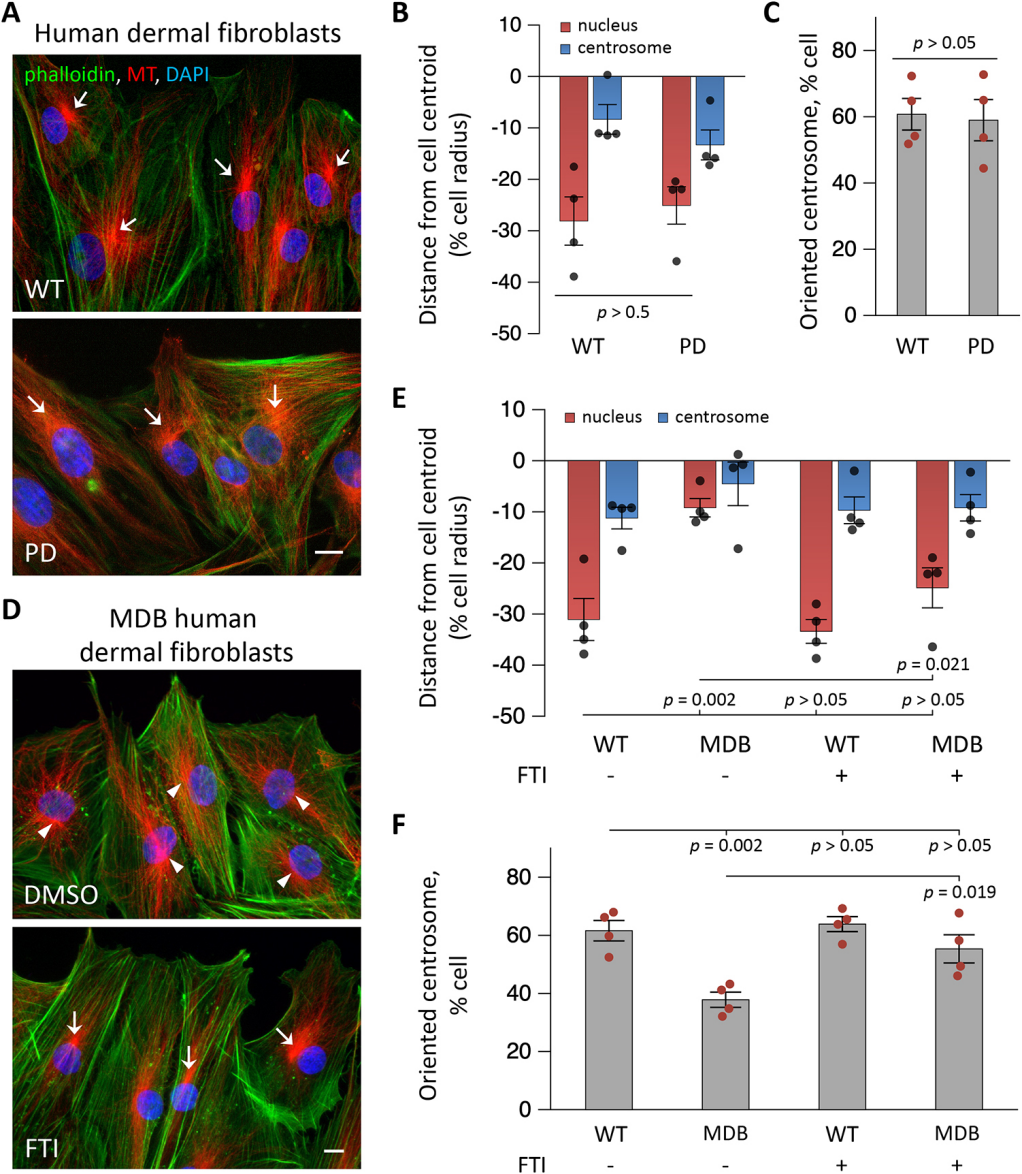
**Nuclear positioning and centrosome orientation in LPA-stimulated human fibroblasts expressing farnesylated prelamin A.** (A) Representative fluorescence images of WT and PD fibroblasts (heterozygous *LMNA* L647R variant) showing similar rearward nuclear positioning and oriented centrosomes (arrows) after lysophosphatidic acid (LPA) stimulation. Cells were labelled for actin filaments (phalloidin), microtubules (MT) and nuclei (DAPI). Scale bar: 10 µm. In panel A and all subsequent panels of fluorescence images, the wound is oriented toward the top of the panel. (B) Quantification of nuclear (red) and centrosomal (blue) positions along the front–back axis of LPA-stimulated wild-type (WT) and PD fibroblasts. The cell centroid is defined as ‘0’, plus (+) values are toward the leading edge, and minus (−) values are toward the cell rear. (C) Quantification of centrosome orientation for the cells treated as in B. Random centrosome orientation is 33%. (D) Representative fluorescence images of MDB fibroblasts showing inhibited rearward nuclear positioning and non-oriented centrosomes (arrowheads) after vehicle (DMSO) treatment and improved rearward nuclear positioning and oriented centrosomes (arrows) after FTI treatment (bottom, arrows). Cells were labelled for actin filaments (phalloidin), microtubules and nuclei (DAPI). Scale bar: 10 µm. (E) Quantification of nuclear (red) and centrosomal (blue) positions along the front–back axis of LPA-stimulated WT and MDB fibroblasts treated without or with FTI. (F) Quantification of centrosome orientation for the cells treated as in E. Values are means±s.e.m. from *N*=4 biological replicates (*n*=30 cells per experiment for B,E, and *n=*75 cells per experiment for C,F). *P-*values were calculated by either unpaired two-tailed Student's *t*-test (B,C) or one-way ANOVA with post hoc Tukey's test (E,F).

LPA-stimulated PD fibroblasts, which expressed farnesylated prelamin A and lamin A in a roughly 1:1 ratio, did not demonstrate a polarity defect (Fig. 2A–C). In contrast, in MDB fibroblasts, which expressed significantly more farnesylated prelamin A than lamin A, rearward nuclear positioning was defective, resulting in improper centrosome orientation and defective cell polarity; FTI treatment rescued these defects (Fig. 2D–F). These results suggest that farnesylated prelamin A and the farnesylated unprocessed variant cause defective cell polarity in a farnesylation- and dosage-dependent manner.

### Polarization of migratory mouse fibroblasts expressing farnesylated prelamin A

**Fig. 3.**
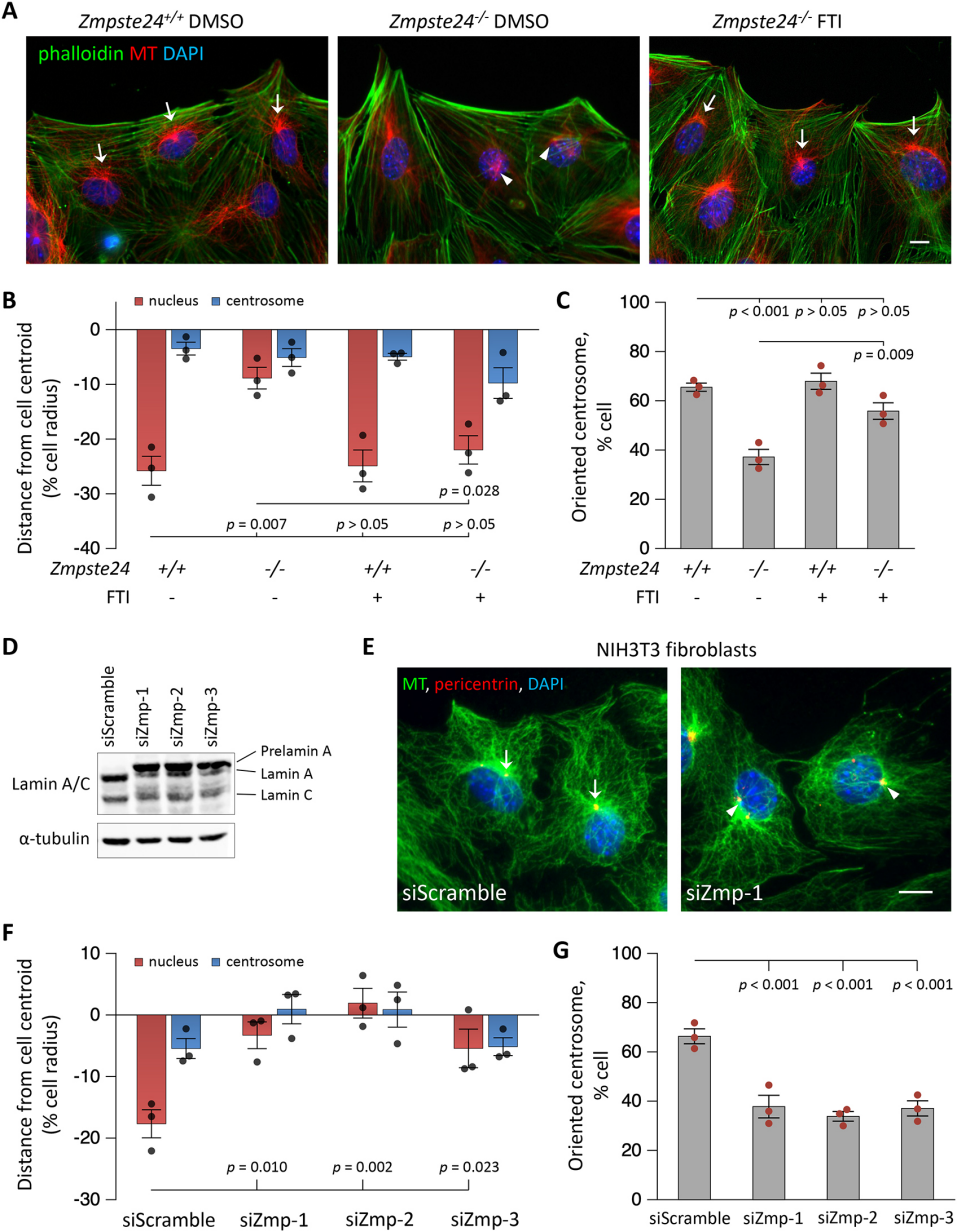
**Nuclear positioning and centrosome orientation in LPA-stimulated *Zmpste24*^+/+^ and *Zmpste24^−/−^* MEFs and NIH3T3 fibroblasts depleted of ZMPSTE24.** (A) Representative fluorescence images of *Zmpste24^+/+^* and *Zmpste24^−/−^* MEFs showing normal rearward nuclear positioning and oriented centrosomes (left, arrows) in vehicle (DMSO)-treated *Zmpste24^+/+^* MEFs, impaired rearward nuclear positioning and non-oriented centrosomes (middle, arrowheads) in vehicle-treated *Zmpste24^−/−^* MEFs, and improved nuclear positioning and oriented centrosomes (arrows) in *Zmpste24^−/−^* MEFs treated with FTI (right, arrows). Cells were labelled for actin filaments (phalloidin), microtubules (MT) and nuclei (DAPI). Scale bar: 10 μm. (B) Quantification of nuclear (red) and centrosomal (blue) positions along the front–back axis of LPA-stimulated *Zmpste24*^+/+^ and *Zmpste24*^−/−^ MEFs treated without or with FTI. (C) Quantification of centrosome orientation for the cells treated as in B. (D) Representative immunoblots showing lamin A and prelamin A in extracts from NIH3T3 fibroblasts treated with scrambled siRNA (siScramble) and three siRNAs targeting *Zmpste24* (siZmp-1–3). Note the accumulation of prelamin A in cells with knockdown of ZMPSTE24. Blots were probed with antibodies to lamin A/C (recognizing prelamin A, lamin A and lamin C) and to tubulin as a loading control. Blots represent three independent experiments. (E) Representative fluorescence images of NIH3T3 fibroblasts labelled for microtubules (MT), centrosomes (pericentrin) and nuclei (DAPI) showing normal rearward nuclear positioning and oriented centrosomes (arrows) in fibroblasts treated with a scrambled siRNA, and impaired rearward nuclear positioning and non-oriented centrosomes (arrowheads) in fibroblasts treated with an siRNA targeting *Zmpste24*. Scale bar: 10 μm. (F) Quantification of nuclear and centrosomal positions in LPA-stimulated NIH3T3 fibroblasts treated with scrambled siRNA or three different siRNAs targeting *Zmpste24*. (G) Quantification of centrosome orientation for the cells treated as in F. Values are means±s.e.m. from *N*=3 biological replicates (*n*=90 cells). *P*-values were calculated by one-way ANOVA with a post hoc Tukey's test (B,C) or Dunnett's test (F,G). In A,E, the wound is at the top.

Unlike humans with total loss-of-function *ZMPSTE24* variants, which lead to restrictive dermopathy and perinatal death, *Zmpste24*^−/−^ mice appear normal until 4 weeks of age, and then develop progeroid phenotypes and die at 5–6 months (Navarro et al., 2005; Pendás et al., 2002; Bergo et al., 2002). These differences prompted us to also examine cell polarization in *Zmpste24*^−/−^ MEFs. In LPA-stimulated wounded monolayers of *Zmpste24*^−/−^ MEFs, centrosome orientation and rearward nuclear positioning were significantly impaired, and FTI treatment restored near-normal nuclear positioning and improved centrosome orientation in these cells (Fig. 3A–C).

To confirm that defective cell polarization was caused by loss of ZMPSTE24, we used siRNAs to deplete the protein from NIH3T3 fibroblasts. Because reliable antibodies for detection of ZMPSTE24 by immunoblotting were unavailable, we used the level of prelamin A to assess knockdown. Treatment with three different *Zmpste24* siRNAs reduced levels of lamin A and increased prelamin A (Fig. 3D). When stimulated with LPA, cells treated with the *Zmpste24* siRNAs all failed to undergo nuclear movement and centrosome orientation, whereas these measures were normal in cells treated with a scrambled siRNA (Fig. 3E–G).

### Prelamin A inhibits cell polarization in a dosage-dependent manner

**Fig. 4.**
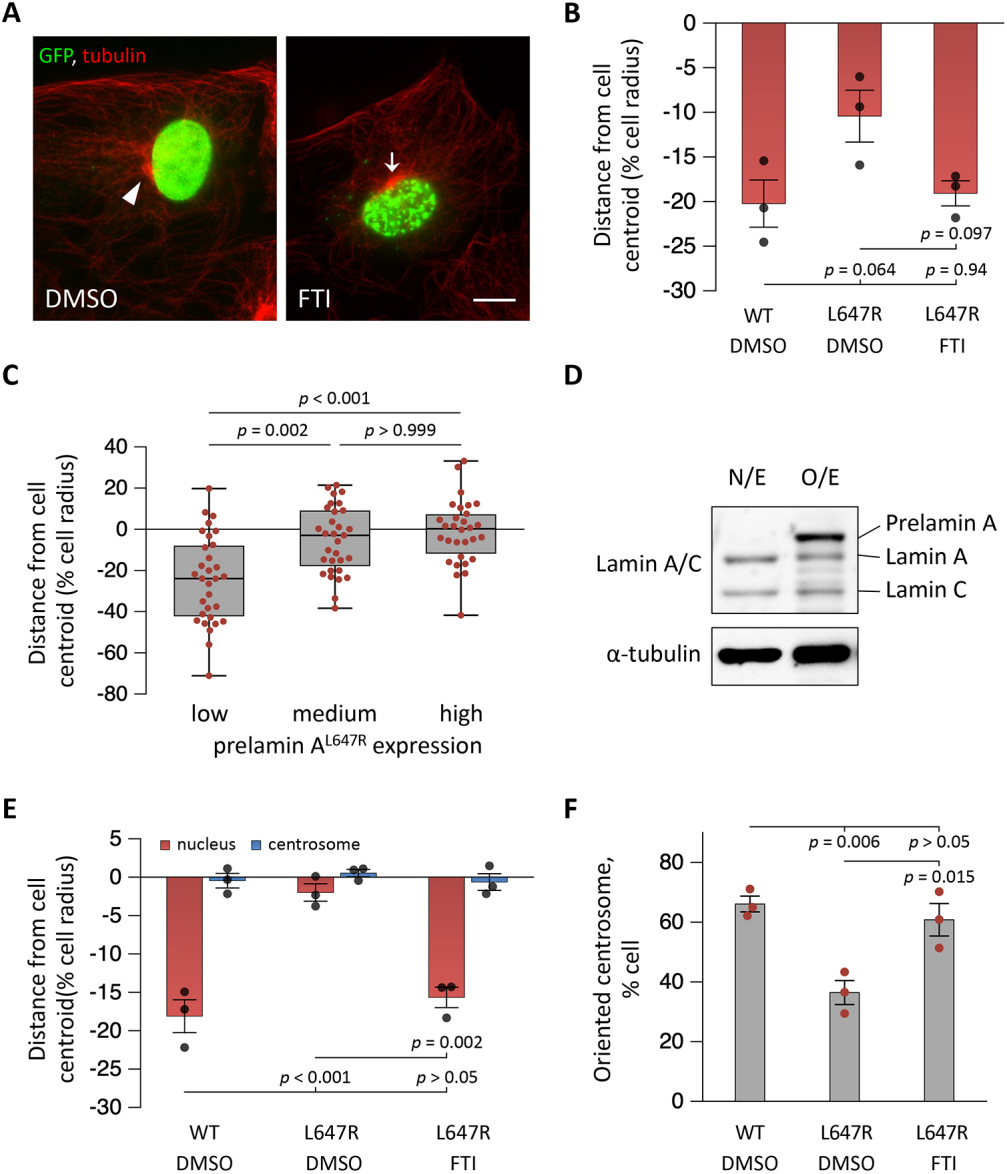
**Nuclear positioning and centrosome orientation in NIH3T3 fibroblasts expressing EGFP-tagged lamin A or prelamin A L647R.** (A) Representative fluorescence images showing EGFP–prelamin A L647R expression in cells pretreated with vehicle control (DMSO) or 2.5 µM FTI-277 (FTI). Arrowhead, non-oriented centrosome; arrow, oriented centrosome. Scale bar: 10 μm. (B) Quantification of nuclear positions of LPA-stimulated NIH3T3 fibroblasts expressing EGFP-tagged lamin A or prelamin A L647R treated with the DMSO vehicle control or 2.5 µM FTI-277 as indicated. (C) Box plot of nuclear positions in LPA-stimulated NIH3T3 fibroblasts expressing low, medium and high levels of EGFP–prelamin A L647R. Each dot represents the relative nuclear position of a cell. Boxes show the interquartile range, whiskers show the full range, and the median is marked with a line. (D) Immunoblot showing the expression of lamin A/C species in NIH3T3 fibroblasts stably overexpressing prelamin A L647R (O/E) and in non-expressing (N/E) control fibroblasts. Blots were probed with antibodies to lamin A/C (recognizing prelamin A, lamin A and lamin C) and to tubulin as a loading control. Blots represent three independent experiments. (E) Quantification of nuclear (red) and centrosomal (blue) positions in LPA-stimulated NIH3T3 fibroblasts expressing either mature lamin A (WT) or prelamin A L647R treated with DMSO or with FTI. (F) Quantification of centrosome orientation for the cells treated as in E. Values are mean±s.e.m. from *N*=3 biological replicates (*n*=75 cells). *P*-values were calculated by Kruskal–Wallis analysis with post hoc Dunn's test (C) or one-way ANOVA with post hoc Tukey's test (B,E,F).

To test whether acute expression of the non-cleavable prelamin A variant affected cell polarization, we expressed it in NIH3T3 fibroblasts by microinjecting plasmid DNAs encoding EGFP-tagged lamin A (control) or prelamin A L647R into cells pretreated with DMSO or 2.5 µM FTI-277 for 24 h. Cell polarization was then stimulated by LPA 1 h after injection, which was sufficient time for expression of the injected plasmid. There was a trend towards reduced rearward nuclear movement with expression of prelamin A L647R and reversal by FTI pretreatment; however, the differences did not reach statistical significance (Fig. 4A,B). Given that the cell polarity defects in PD and MDB fibroblasts correlated with levels of prelamin A expression, we speculated that the polarity-inhibiting effect of prelamin A was dosage dependent. We therefore segregated EGFP–prelamin A L647R-expressing cells into three groups according to their levels of EGFP–prelamin A L647R expression (see Materials and Methods). In cells with a relatively low level of prelamin A L647R expression, nuclei demonstrated normal rearward positioning, whereas in cells with high expression, nuclei failed to be positioned rearward and instead resided near the cell center (Fig. 4C). Thus, farnesylated prelamin A L647R inhibits nuclear movement and cell polarization in a dosage-dependent manner. We also established NIH3T3 fibroblasts that stably express an elevated level of Myc-tagged prelamin A L647R (Fig. 4D). Both rearward nuclear movement and centrosome orientation were inhibited in these cells and restored by FTI treatment (Fig. 4E,F).

### Polarization defects caused by farnesylated prelamin A are mediated by SUN1 and the microtubule cytoskeleton

**Fig. 5.**
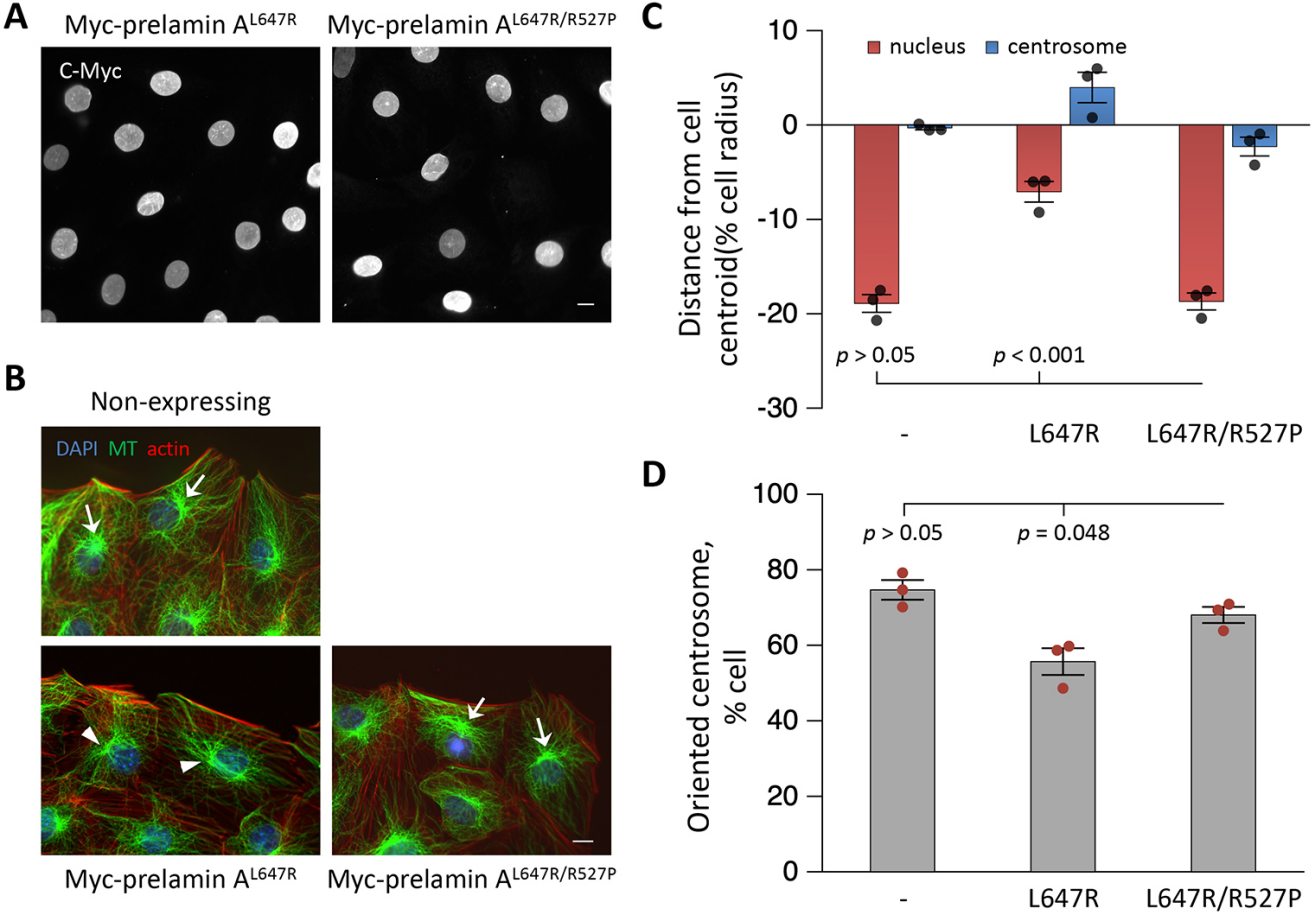
**Nuclear positioning and centrosome orientation in NIH3T3 fibroblasts expressing Myc–prelamin A L647R or Myc–prelamin A L647R/R527P.** (A) Representative images of NIH3T3 fibroblasts expressing the indicated Myc-tagged prelamin A proteins and stained for the Myc tag to show expression levels and nuclear localization of the proteins. (B) Representative images of cells expressing the indicated proteins after LPA stimulation and stained for microtubules (MT), actin filaments (phalloidin) and nuclei (DAPI). Arrowheads indicate non-oriented and arrows indicate oriented. (C) Quantification of nuclear (red) and centrosomal (blue) positions for cells treated as in B. (D) Quantification of centrosome orientation for the cells treated as in B. Scale bars: 10 µm. Values are mean±s.e.m. from *N*=3 biological replicates (*n*=90 cells). *P*-values were calculated by one-way ANOVA with post hoc Tukey's test.

Polarization defects in fibroblasts expressing progerin are caused in part by elevated association between microtubules and the nucleus through elevated SUN1 (Chang et al., 2019). We recently found that disrupting the interaction of progerin with SUN1 by introducing the point mutation R527P into progerin abolishes its ability to disrupt normal nuclear movement and centrosome orientation (Li et al., 2026). We introduced the same R527P point mutant into prelamin A L647R and expressed it in NIH3T3 fibroblasts, in which it was expressed at levels equivalent to those of prelamin A WT and localized to the nucleus as progerin (Fig. 5A). In wounded monolayer experiments, fibroblasts expressing this double mutant exhibited normal nuclear movement and centrosome orientation, strongly suggesting that prelamin A functions similarly to progerin (Fig. 5B–D).

**Fig. 6.**
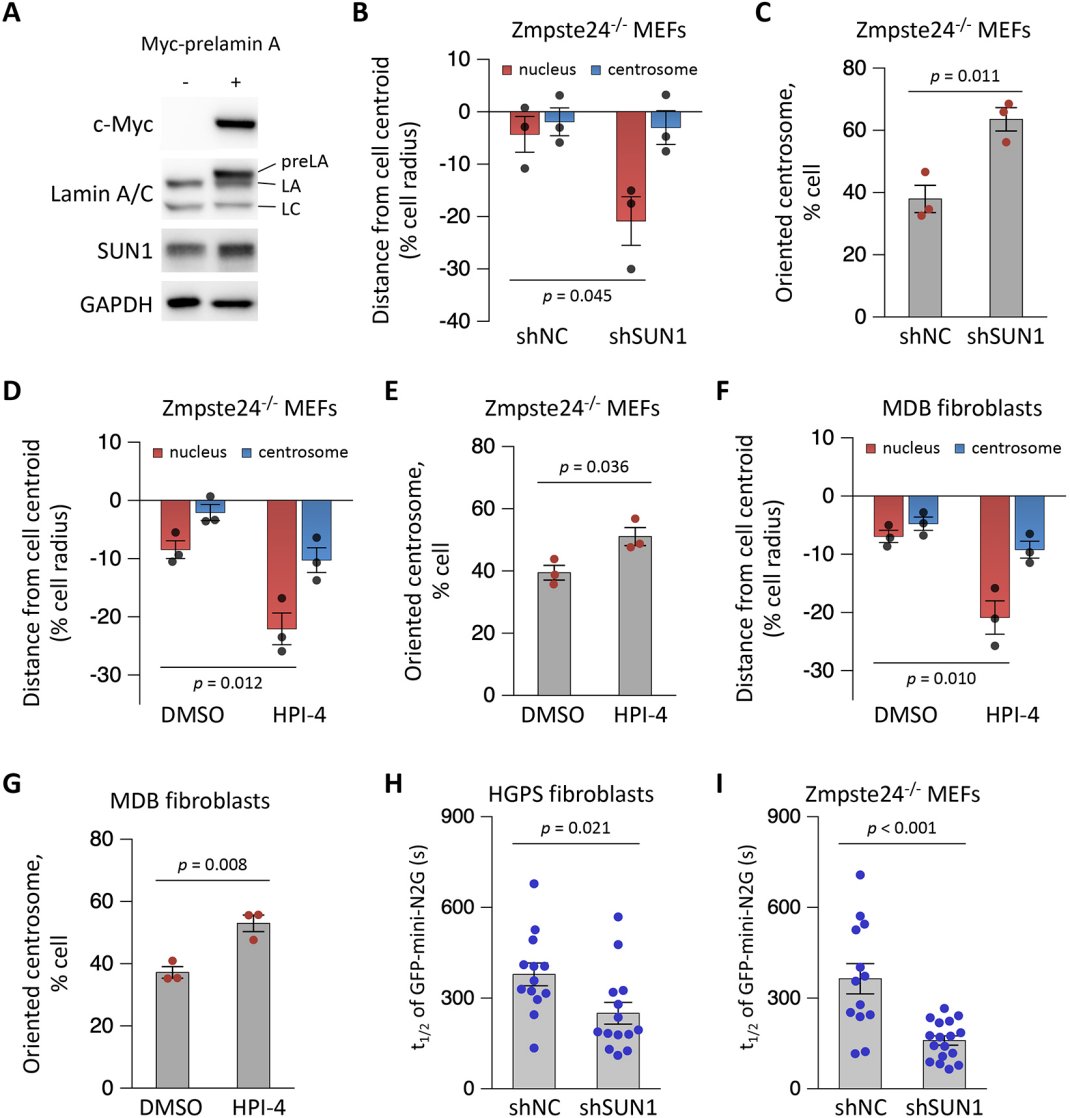
**Nuclear positioning and centrosome orientation in prelamin A-expressing cells treated with shRNA against SUN1 or the dynein inhibitor HPI-4.** (A) Immunoblots showing the expression level of SUN1 in NIH3T3 fibroblasts expressing Myc–prelamin A L647R. Blots were probed with antibodies to Myc, lamin A/C and SUN1, and GAPDH as a loading control. preLA, prelamin A; LA, lamin A; LC, lamin C. (B) Quantification of nuclear (red) and centrosomal (blue) positions of LPA-stimulated *Zmpste24*^−/−^ MEFs expressing noncoding control (shNC) or SUN1-specific (shSUN1) shRNAs. See Fig. S5A for SUN1 levels. (C) Quantification of centrosome orientation for the cells treated as in panel B. (D) Quantification of nuclear (red) and centrosomal (blue) positions of LPA-stimulated *Zmpste24*^−/−^ MEFs pretreated with the vehicle control (DMSO) or 10 µM HPI-4 for 1 h. (E) Quantification of centrosome orientation for the cells treated as in D. (F) Quantification of nuclear (red) and centrosomal (blue) positions along the front–back axis of LPA-stimulated MDB fibroblasts pretreated with the vehicle control (DMSO) or 10 µM HPI-4 for 1 h. (G) Quantification of centrosome orientation for the cells treated as in panel F. (H,I) Half times (*t*_1/2_) of FRAP for EGFP–mini-N2G in HGPS fibroblasts (H) and *Zmpste24*^−/−^ MEFs (I) treated with noncoding (shNC) or SUN1-specific (shSUN1) shRNAs. Values are mean±s.e.m. from *N*=3 biological replicates (B–G, *n*=90 cells; H,I, *n*≥13 cells). *P*-values were calculated by unpaired two-tailed Student's *t*-test.

The polarization defects in progerin-expressing fibroblasts can be ameliorated by reducing the expression of SUN1 or by treating with an inhibitor (HPI-4) of the microtubule motor dynein (Chang et al., 2019). First, we confirmed that, similar to progerin expression (Chen et al., 2012; Chang et al., 2019), stable expression of prelamin A (L647R) led to an increase in SUN1 levels (Fig. 6A). We then tried the same treatments used to rescue the defects in progerin-expressing fibroblasts to determine whether they would affect the cell polarity defects in *Zmpste24*^−/−^ MEFs. Depletion of SUN1 by shRNA rescued both defective nuclear positioning and centrosome orientation in *Zmpste24*^−/−^ MEFs (Fig. 6B,C; Fig. S5A). Likewise, HPI-4 inhibition of dynein, which does not affect nuclear positioning or centrosome orientation in WT fibroblasts (Chang et al., 2019), rescued both parameters in *Zmpste24*^−/−^ MEFs (Fig. 6D,E). HPI-4 treatment also reversed the cell polarization defects in MDB fibroblasts (Fig. 6F,G).

Given the rescued polarity defects in prelamin A-expressing cells and in our earlier study of progerin-expressing cells (Chang et al., 2019), we were curious whether SUN1 depletion would also rescue the reduced diffusional mobility of nesprin-2 in these cells. Strikingly, depletion of SUN1 increased the diffusional mobility of mini-N2G in both *Zmpste24^−/−^* MEFs and HGPS fibroblasts (Fig. 6H,I; Fig. S5B–D). These results indicate that both progerin and farnesylated prelamin A inhibit cell polarity through the same SUN1- and microtubule-dependent mechanism and that the reduced mobility of mini-N2G is due to SUN1.

### Expression of carboxyl-terminal tail fragments of prelamin A and progerin are sufficient to inhibit cell polarity when farnesylated

**Fig. 7.**
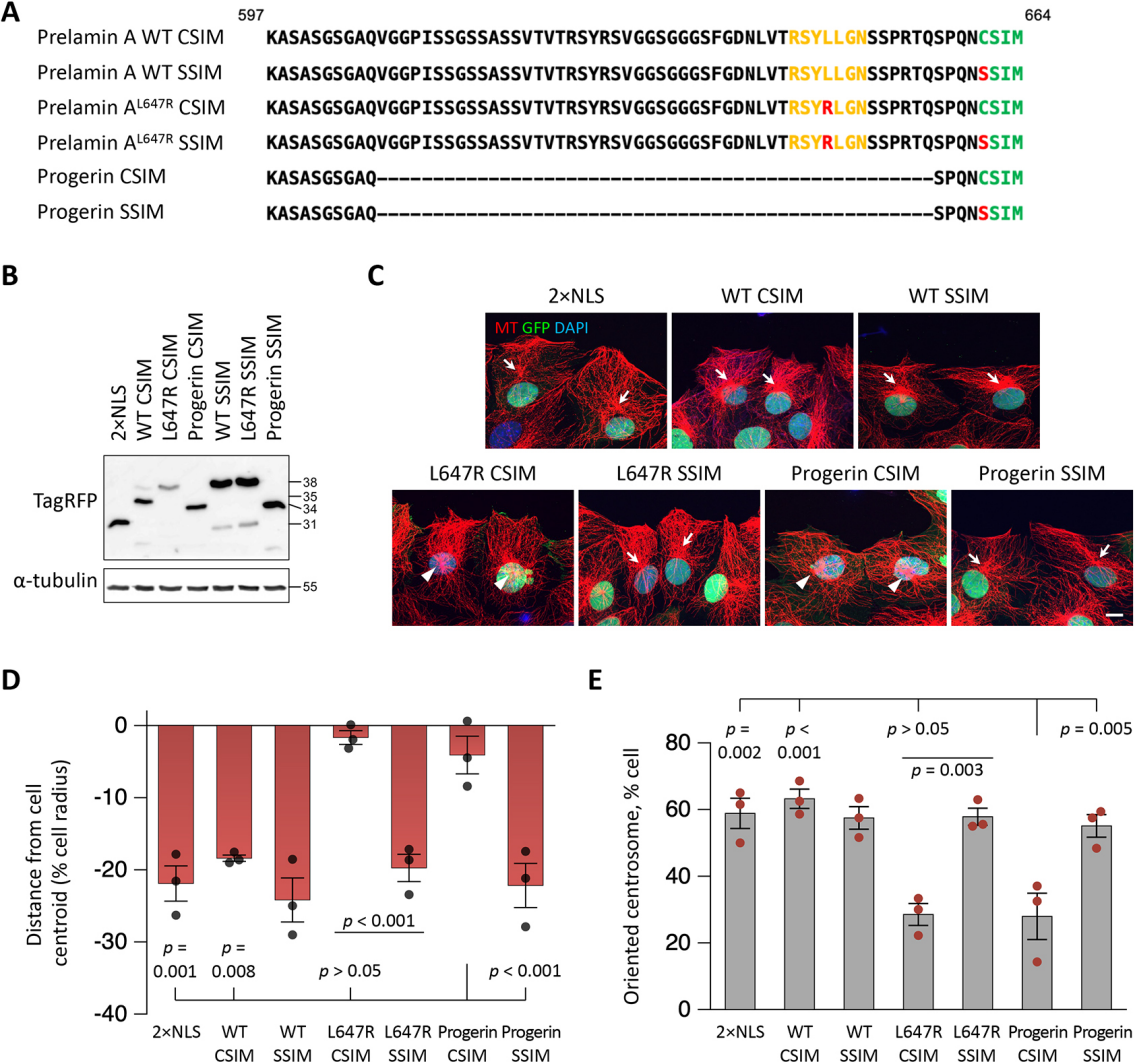
**Carboxyl-terminal tails of prelamin A and variants exhibit farnesylation-dependent inhibition of cell polarity.** (A) Sequences of the tails of WT prelamin A and its variants. The farnesylation motif CSIM is in green. Replacement of the cysteine with serine (red) prevents farnesylation. The dashed lines indicate the deleted amino acids in progerin. The *ZMPSTE24* cleavage site is in yellow with the L647R variant in red. All fragments are 2×NLS-tagged. (B) Immunoblots of NIH3T3 fibroblast 48 h after expression of the indicated TagRFP-tagged lamin A tail constructs. Blots were probed with antibodies to TagRFP or tubulin as a loading control. Molecular masses (kDa) of the major expressed bands are shown on the right. Blots represent three independent experiments. (C) Representative fluorescence images of LPA-stimulated fibroblasts 24 h after doxycycline induction of the indicated EGFP–prelamin A tail constructs and stained for microtubules (MT), EGFP and nuclei (DAPI) showing nuclear positioning and centrosome orientation. Arrows, oriented centrosomes; arrowheads, unoriented centrosomes. Scale bar: 20 µm. (D) Quantification of nuclear positions in LPA-stimulated NIH3T3 fibroblasts expressing the indicated lamin tails. (E) Quantification of centrosome orientation for the cells treated as in D. Values are mean±s.e.m. from *N=*3 biological replicates (*n*=90 cells). *P*-values were calculated by one-way ANOVA with Tukey's test.

The protein sequences of prelamin A WT, prelamin A L647R and progerin differ only in their extreme carboxyl-termini (Fig. 7A). Therefore, we tested whether the cell polarity defects caused by the prelamin A variants are mediated by this region. We fused their carboxyl-termini tails (hereafter ‘tails’) with two nuclear localization sequences (2×NLS) and a fluorescent protein (either TagRFP or mScarlet-I) to drive localization to the nucleus and allow visualization of expression. To determine whether farnesylation contributed to the effects of these tails, we expressed both a WT construct harboring a cysteine-serine-isoleucine-methionine (CSIM) sequence at its C-terminus and a non-farnesylatable variant in which CSIM was replaced with SSIM. Immunoblots of whole-cell lysates revealed proteins of the expected size (Fig. 7B). The prelamin A WT CSIM tail migrated as two bands, a minor one at the size predicted for an unprocessed tail (38 kDa) and a major one at a size predicted for the processed form (35 kDa). Consistent with this, the unfarnesylated prelamin WT SSIM tail, which cannot be processed, migrated predominantly as a higher molecular mass species (38 kDa). Both the CSIM and SSIM versions of the prelamin A L647R and progerin tails remained mostly unprocessed, although we detected variable amounts of lower molecular mass species (Fig. 7B). All constructs localized to the nucleus and confocal imaging showed that the prelamin A L647R and progerin CSIM tails showed localization to the nuclear periphery, whereas the cleavable WT CSIM prelamin A tail and the SSIM tails were predominantly nucleoplasmic (Fig. 7C; Fig. S6A). Inducible expression of EGFP-tagged versions of the prelamin A L647R or progerin CSIM tails, but not that for prelamin A WT tails, blocked rearward nuclear positioning and centrosome orientation (Fig. 7C–E). Non-farnesylatable SSIM tails did not block rearward nuclear positioning or centrosome orientation. Additionally, FTI treatment rescued the inhibition of nuclear movement in cells expressing farnesylatable CSIM tails of prelamin A L647R or progerin (Fig. S6B,C). Thus, farnesylatable forms of the carboxyl-terminal tails of prelamin A L647R and progerin are sufficient to disrupt cell polarity.

## DISCUSSION

A prominent hypothesis for how alterations in lamin A/C promote diseases, including premature aging syndromes, is that they disrupt the normal mechanical behavior of the nucleus. The LINC complex is anchored in the nuclear envelope by the lamina and transduces mechanical inputs to the nucleus. Here, we show that prelamin A with a single amino acid substitution that abolishes the ZMPSTE24 cleavage site (as in PD) or a *ZMSTE24* variant leading to reduced activity (as in MDB) interfered with the mobility of mini-N2G and prevented nesprin-2G- and actin-dependent rearward movement of the nucleus in polarizing fibroblasts. These results are reminiscent of our earlier study showing the same defects with progerin expression (Chang et al., 2019). Critically, in all these cases, prelamin A or its variants maintain the farnesyl moiety, and treatment with FTI reverses the defects in mini-N2G mobility and force coupling to move the nucleus. Also similar to progerin expression (Chang et al., 2019), these farnesylated prelamin A species appear to cause defects in cell polarity by generating excessive nuclear–microtubule interactions through SUN1. Given that the mobility of mini-N2G was reduced in all cells with impaired nuclear movement and that SUN1 knockdown rescued both defects, we suggest that farnesylated prelamin A species enhance nesprin-2G interaction with SUN1, immobilizing and preventing it from engaging SUN2, which is necessary to support the actin functionality of nesprin-2G. The inconsistent effects of prelamin A expression on the mobilities of SUN2 and emerin (only human PD and MDB fibroblasts affected) and their lack of rescue by FTI treatment (Fig. 1C–E), suggest that the altered mobilities of these proteins are not directly related to the polarity phenotypes.

In addition to the proteolytic processing of prelamin A, ZMPSTE24 functions in the clearance of clogged translocons during biosynthesis of secreted proteins. Upon clogging, ZMPSTE24 is recruited to the translocon to cleave the untranslocated protein into fragments that can be degraded by the proteasomes (Ast et al., 2016). In contrast to cells with deficiency of ZMPSTE24, cells expressing the prelamin A L647R variant should not have defective clearance of clogged translocons, suggesting that the nuclear movement and polarity defects are not related to loss of this function. Experiments on the prelamin A L647R variant further show that the polarity defects are indeed caused by the incomplete processing of prelamin A.

Our results suggest a correlation between cell polarity defects and the severity of a progeroid disorder. Cells from a patient with PD with the heterozygous *LMNA* L647R variant expressing the prelamin A variant and lamin A in approximately a 1:1 ratio had no detectable polarity defect. This patient had a relatively mild progeriod syndrome (Wang et al., 2016). Fibroblasts from the patient with MDB homozygous for the *ZMPSTE24* L425P variant expressing mostly farnesylated prelamin A had a polarity defect. Consistently, a patient with this variant has been described with a phenotype that is more severe and more comparable to HGPS (Harhouri et al., 2016). These results also show that cell polarity defects, similar to the severity of disease (Barrowman et al., 2012), correlate with the expression of prelamin A. This might occur because either (1) the presence of adequate quantities of WT lamin A can suppress the inhibitory effects of farnesylated prelamin A or (2) farnesylated prelamin A causes the polarization defect only when its expression reaches a critical level relative to lamin A. We favor the second explanation because reducing the amount of prelamin A in *Zmpste24*^−/−^ mice eliminates disease phenotypes (Fong et al., 2004). Prelamin A also binds to SUN1, which is a key contributor to the defective cell polarization in aged cells (Chang et al., 2019; Chen et al., 2012, 2014; Crisp et al., 2006).

Both the patient with PD with the *LMNA* L647R variant and patients with HGPS are heterozygous for the variant alleles that express the uncleavable prelamin A variant or progerin in roughly a 1:1 ratio with lamin A. However, these different prelamin A variant proteins appear to have different potencies in causing cell polarity defects and disease phenotypes with progerin being more deleterious than prelamin A L647R (Chang et al., 2019; Ullrich and Gordon, 2015; Wang et al., 2016) (with the caveat that only one patient with the *LMNA* L647R variant has been reported in the literature). The molecular mechanism underlying this difference is not yet understood. Our results nonetheless suggest that expression of farnesylated progerin is more deleterious than expression of farnesylated prelamin A. The 50 amino acids present in prelamin A but missing in progerin might be critical for an additional aspect of lamin A function beyond LINC complex-dependent polarization. Alternatively, the presence of these amino acids might partially lessen the harmful effect of the uncleaved carboxyl-terminal end of farnesylated prelamin A. Several *LMNA* variants outside the carboxyl-terminus have been linked to mandibuloacral dysplasia type A and atypical progeroid syndromes (Novelli et al., 2002; Garg et al., 2009). As there is evidence that certain variants outside of the carboxyl-terminal tail affect SUN1 binding, it is possible that alterations in the SUN1–lamin A interaction also contribute to disease etiology in these cases (Haque et al., 2010). Alternatively, such variants might perturb other processes but still generate similar phenotypes. It will be interesting to determine whether these variants also impair the LINC complex and its functions such as nuclear positioning.

To understand the contribution of the 50 amino acid fragment deleted in progerin, we expressed the carboxyl-terminal tails of prelamin A WT, prelamin A L647R and progerin. Both the prelamin A L647R and progerin tails inhibited cell polarization, but only when expressed as farnesylatable (CSIM) versions. We attribute the lack of inhibition of the prelamin A WT tail to the observation that it migrates as a lower molecular mass species than the unfarnesylatable SSIM tail and does not localize to the nuclear rim, suggesting that it has been processed and the farnesyl moiety has been removed. The tail fragments we used did not contain the lamin A/C rod domain, which is responsible for dimerization, or the immunoglobulin-like β-fold motif, which mediates certain protein interactions of lamin A (Haque et al., 2010; Dittmer et al., 2014). As farnesylated prelamin A and progerin have been shown to bind SUN1 more strongly than processed lamin A WT and enhance SUN1 levels in the nuclear envelope (Crisp et al., 2006; Haque et al., 2006, 2010; Chen et al., 2012, 2014; Chang et al., 2019), we favor the interpretation that these farnesylated prelamin A tails, similar to full-length prelamin A, enhance interaction of nesprin-2G, with SUN1 favoring its interaction with microtubules. This in turn would prevent nesprin-2G from interacting with SUN2 to support nesprin-2G interaction with actin filaments. This interpretation is consistent with our observations that the polarity defects are rescued by treatments knocking down SUN1 or inhibiting dynein. It is also possible that these fragments affect the activity of the LINC complex indirectly by inserting their farnesylated moieties into the nuclear membrane.

Our current results and those of a previous study (Chang et al., 2019) support the idea that a common feature of progeroid disorders is alterations in the function of the LINC complex. Rather than a simple loss- or gain-of-function, our data suggest that expression of prelamin A variants biases the LINC complex to function with microtubules at the expense of functioning with actin filaments. Although the resulting defects in cell polarity that we measured might themselves contribute to the progeroid disorders, this change in cytoskeletal preference of the LINC complex is likely to affect the mechanical function of cells in tissues affected in the progeroid syndromes, such as bone, muscle and the vasculature. Our results also indicate that the defects in cell polarity resulting from expression of prelamin A variants can be rescued by SUN1 knockdown or treatment with a dynein inhibitor. Thus, it might be possible to devise treatments for premature aging syndromes that do not require blocking the farnesylation of prelamin A or its variants or removing them. Further studies on the cellular defects induced by the farnesyl moieties could provide insights into the mechanisms of prelamin A-mediated premature aging and help to explain why lamin A is expressed as a precursor that undergoes complicated yet seemingly unnecessary processing.

## MATERIALS AND METHODS

### Reagents

LPA was purchased from Avanti Polar Lipids and Alexa Fluor Plus 555 Phalloidin (A30106) and FITC Phalloidin (F432) were from Thermo Fisher Scientific. FTI-277 was from Sigma-Aldrich. Rat anti-prelamin A antibody (Lee et al., 2010) was provided by Dr Loren Fong and Dr Stephen Young, University of California, Los Angeles (UCLA) [1:3000 for western blotting (WB)]. Rabbit polyclonal anti-lamin A/C antibody was from Santa Cruz Biotechnology (sc-20681; 1:5000 for WB). Mouse monoclonal anti-GAPDH antibody was from Thermo Fisher Scientific (AM4300; 1:3000 for WB) and BioLegend (#607902; 1:1000 for WB). Rat monoclonal anti-tyrosinated α-tubulin antibody was from European Collection of Animal Cell Cultures [clone YL1/2; 1:20 for immunofluorescence (IF) and 1:1000 for WB]. Mouse monoclonal anti-pericentrin antibody was from BD Biosciences (#611814; 1:400 for IF). Mouse monoclonal anti-SUN1 antibody was provided by Dr Brian Burke, Agency for Science, Technology and Research (A*STAR), Singapore (1:1000 for WB). Mouse monoclonal anti-Myc antibody (sc-40; 1:1000 for WB, 1:200 for IF) and mouse monoclonal anti-β-actin antibody (sc-47778; 1:4000 for WB) were from Santa Cruz Biotechnology. Mouse monoclonal anti-TagRFP antibody was from Kerafast (EFH005; 1:2000 for WB). Horseradish peroxidase-conjugated secondary antibodies were from GE Healthcare (dilution 1:5000). All secondary dye-conjugated antibodies were from Jackson ImmunoResearch (dilution 1:200). Unless otherwise noted, all other chemicals were from Sigma-Aldrich.

### Cells

Dermal fibroblasts from a 5.8-year-old boy homozygous for the *ZMPSTE24* c.1274T>C (p.L425P) variant (cell line #PSADFN373) and WT control fibroblasts (cell line #PSFDFN376) were obtained under the Material Transfer Agreement from the Progeria Research Foundation. We previously described the dermal fibroblasts from a 17-year-old woman heterozygous for the *LMNA* T>G transversion at nucleotide c.1940T>G (p.L647R) variant, which were obtained with approval of the Columbia University Irving Medical Center Institutional Review Board (Wang et al., 2016). WT human fibroblasts (cell line #HGFDFN168) and fibroblasts from a patient with HGPS (cell line #HGADFN167) were also obtained under the Material Transfer Agreement from the Progeria Research Foundation. Dr Loren Fong and Dr Stephen Young (UCLA), and Dr Susan Michaelis (Johns Hopkins University) provided fibroblasts from *Zmpste24^+^*^/+^ and *Zmpste24*^−/−^ mouse embryos, which have been previously described (Bergo et al., 2002; Leung et al., 2001). Mouse NIH3T3 fibroblasts were from American Type Culture Collection. All the cell lines were regularly tested for contamination.

Human fibroblasts were cultured in Dulbecco's modified Eagle medium (DMEM, Corning) containing 15% fetal bovine serum (Gemini). MEFs were cultured in DMEM containing 10% fetal bovine serum. NIH3T3 fibroblasts and HEK293T cells were cultured in DMEM with 10% calf serum (Gemini). All media were supplemented with 10 mM HEPES (pH 7.4), penicillin and streptomycin (Thermo Fisher Scientific). All cells were cultured with 5% CO_2_ at 37°C.

### siRNA transfection

siRNA oligonucleotides were from Shanghai GenePharma and transfection was performed using Lipofectamine RNAiMAX (Thermo Fisher Scientific) according to the manufacturer's protocol. The siRNA sequences used were: siScramble, 5′-UUCUCCGAACGUGUCACGU-3′; siZmp-1, 5′-GGAGUCUGUACAAUACUUUUG-3′; siZmp-2, 5′-AAGGUGUAUGUUGUUGAAGGA-3′; and siZmp-3, 5′-AUGAGGUUCUGUCUUUCUGCC-3′. The shRNAs used have been previously described (Zhu et al., 2017). To generate NIH3T3 fibroblasts with stable overexpression, prelamin A L647R and lamin A cDNAs were cloned into pMSCV-Myc-puro (Antoku et al., 2019). All constructs were verified by DNA sequencing.

### DNA microinjection

Microneedles were prepared by pulling glass micropipettes with a Sutter P-87 puller. Plasmid DNA (10 μg/ml in 140 mM KCl, 10 mM HEPES, pH 7.4) was loaded into the microneedles and pressure-microinjected into nuclei of cells at the wounded edge of serum-starved monolayers. After 1 h of protein expression, cells were stimulated with 10 μM LPA to induce nuclear movement.

### Expression of prelamin A WT and variant carboxyl-terminal tails

DNA sequences encoding the carboxyl-termini of prelamin A, progerin and prelamin A L647R and their carboxyl-terminus CSIM-to-SSIM amino acid substitutions were constructed from the cDNAs encoding prelamin A and its variants, which were previously described (Wang et al., 2012). These cDNAs were inserted into non-viral pEF1α-2×NLS-mScarlet-I-C4 [pEF1a (Antoku, et al., 2019); 2×NLS-mScarlet-C4 was made by adding 2×NLS to mScarlet-I-C4 from Addgene #85044], lentiviral pLV-EF1α-2×NLS-TagRFP-C4 IRES-blast (pLV-EF1a IRES-blast was from Addgene #85133, and 2×NLS-TagRFP-C4 was made by adding 2×NLS to TagRFP from Evrogen) and lentiviral pInducer100 EGFP-C4 (puromycin) (derived by adding EGFP to pInducer20, Addgene #44012) plasmids. These lentiviral plasmids were transfected into HEK293T cells to make lentiviral particles, which were then used to infect NIH3T3 fibroblasts. The infected cells were selected either with 4 µg/ml blastocidin (GoldBio, B-800) or 1.5 µg/ml puromycin (GoldBio, P-600). The cells infected with pInducer100 were treated with 50–200 ng/ml of doxycycline (Sigma-Aldrich, D9891) prior to the experiment to induce the expression of the desired protein.

### Protein isolation and immunoblotting

Proteins were extracted from cells with 8.55 M urea, 10 mM Tris-HCl (pH 8.0), 2% β-mercaptoethanol, 1 mM phenylmethylsulfonyl fluoride, 1 mM NaF, 10 µM ethylenediaminetetraacetic acid and 1% protease inhibitor cocktail, and separated by electrophoresis in SDS-polyacrylamide slab gels (Thermo Fisher Scientific). Proteins were transferred to nitrocellulose membranes by electroblotting and analyzed by immunoblotting as described previously (Wang et al., 2012). Signals were detected using SuperSignal West Pico PLUS Chemiluminescent Substrate (Thermo Fisher Scientific) and autoradiography film (LabScientific). Immunoblots were scanned with a digital scanner, and the band signal intensities were quantified using Fiji/ImageJ (https://imagej.net/Fiji) and analyzed using Excel (Microsoft). See Fig. S7 for uncropped immunoblots.

### FRAP

Fibroblasts were cultured on chambered coverglasses and transfected using Lipofectamine PLUS (Life Technologies) following the manufacturer's instructions. Plasmids encoding EGFP–mini-N2G, EGFP–SUN2 and EGFP–emerin have been described previously (Luxton et al., 2010; Östlund et al., 2009). To test the effect of farnesylation, 2.5 µM FTI-277 was added to the culture medium 24 h before transfection, for a total treatment duration of 72 h. An equal volume of DMSO was added to the medium as a control. Experiments were performed in chambers with a 5% CO_2_ overlay at 37°C on a Nikon A1R MP multiphoton confocal microscope controlled by NIS-Elements imaging software. A selected area of the same size on each nucleus was photobleached and the fluorescence recovery was monitored at 3 s intervals for the first 30 images and 5 s intervals for another 70 images. The relative intensity of the fluorescence signal was measured in the region of interest using Fiji and normalized to the change in total fluorescence as *I*_rel_=*T*_0_*I*_t_/*T*_t_*I*_0_, where *I*_rel_ is the relative intensity, *T*_0_ is the total cellular intensity before bleaching, *I*_t_ is the average intensity within the bleached region at time point *t*, *T*_t_ is the total cellular intensity at time point *t*, and *I*_0_ is the average intensity within the bleached region before bleaching, as described previously (Östlund et al., 2009). Mean normalized fluorescence plus or minus standard deviation was calculated and plotted versus time after bleaching using Microsoft Excel. A modified time of half fluorescence recovery value (*t*_1/2_) was calculated as *t*_1/2_=ln2×(−1/slope) using data taken from the first 30 s after bleaching.

### Assay of cell polarization

Centrosome orientation and nucleus and centrosome positioning at the wound edge in fixed and stained cells were determined as previously described (Chang et al., 2016). Briefly, monolayers of cells were washed three times with DMEM and incubated in DMEM for 2 days (MEFs and NIH3T3 fibroblasts) or 4 days (human fibroblasts). Serum-starved cells were wounded and allowed to recover for 30 min before addition of 10 μM LPA. Cells were fixed in 4% paraformaldehyde (Electron Microscopy Sciences) after 2 h, permeabilized in 0.3% Triton X-100 in phosphate-buffered saline and stained with 4′,6-diamidino-2-phenylindole (DAPI), fluorescently tagged phalloidin and antibodies against pericentrin and/or tubulin. Images of cells at the wound edge were acquired with a 40× Plan-Apo objective (NA 1.0) on either a Nikon TE300 microscope with a CoolSNAP HQ CCD camera controlled by Metamorph (Molecular Devices), or a Nikon Eclipse Ti microscope with an iXon X3 CCD and a Nikon Qi2 camera controlled by NIS-Elements. Images were analyzed using CellPlot as previously described (Chang et al., 2016). For FTI treatment, 2.5 μM FTI-277 (or DMSO as vehicle control) was added 2 days before LPA stimulation, and the medium (with FTI) was changed daily. For HPI-4 treatment, 10 μM HPI-4 was added 1 h before LPA stimulation.

For Fig. 4C, total GFP intensity of the nucleus was measured for each cell after background subtraction. The normalized intensities (in arbitrary units) were calculated by dividing the total GFP intensities by a constant factor (10,000,000). Cells were then divided into three groups according to the normalized intensities for further analysis: low (intensity<1, *n*=31 cells), medium (1<intensity<2, *n*=31 cells) and high (2<intensity<15, *n*=30 cells).

### Statistics

FRAP data were analyzed and plotted using Excel. Statistical comparisons between two groups were determined using unpaired two-tailed Student's *t*-tests in Excel. Statistical comparison between three or more groups was determined using one-way ANOVA followed by either Tukey's or Dunnett's tests in GraphPad Prism. Statistical comparisons in Fig. 4C were determined in GraphPad Prism using the Kruskal–Wallis test and Dunn's multiple comparisons test. Plots were generated with either Excel or DotPlot (https://changlab.fhs.um.edu.mo/software/dotplot/).

## Supplementary Material

10.1242/joces.264488_sup1 Supplementary information
