# Supplementary material for: Farnesylated prelamin A induces fibroblast polarity defects in premature aging disorders by inhibiting nesprin-2–SUN2 LINC complex function
Source: J Cell Sci. 2026 May 18;139(12):jcs264488. doi: 10.1242/jcs.264488 (PMC13282567; doi:10.1242/jcs.264488)
Supplement: Supplementary information [file joces-139-264488-s1.pdf]

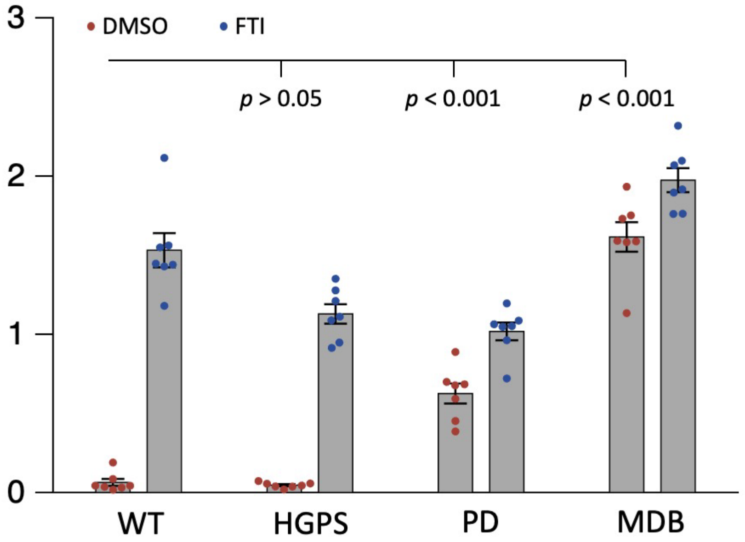

Fig. S1. Quantification of prelamin A in fibroblasts from patients and control. Histogram showing levels of prelamin A detected by immunoblotting whole cell extracts of WT, HGPS, PD and MDB fibroblasts. Values are means ± SEM (*n* = 7 immunoblots) by 1-way ANOVA with Tukey's test.

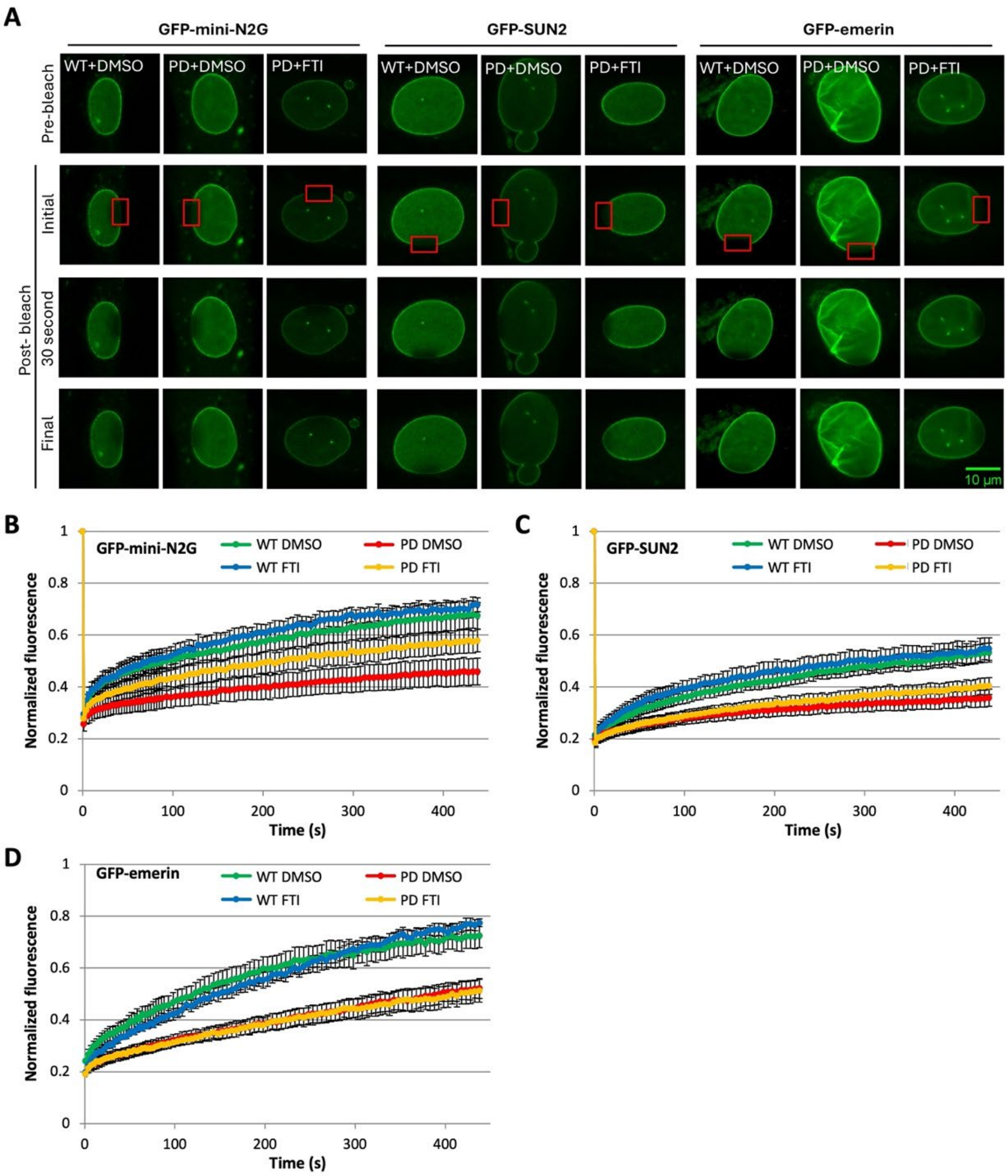

Fig. S2. Diffusional mobilities of mini-N2G, SUN2 and emerin in the nuclear envelope of PD fibroblasts. (A) Representative confocal fluorescence images of WT and PD fibroblasts expressing EGFP-tagged mini-N2G, SUN2 or emerin treated with FTI or DMSO (control) and subjected to FRAP. Images represent nuclear GFP fluorescence pre-bleach and at different times post-bleach as indicated. Final is 442 seconds post photobleaching. (B-D) Normalized FRAP intensities of EGFP-tagged mini-N2G (B), SUN2 (C) and emerin (D) in the nuclear envelope of WT and PD fibroblasts treated with DMSO (control) or FTI-277 as indicated. Values are means ± SEM from $N = 3$ biological replicates.

Journal of Cell Science • Supplementary information

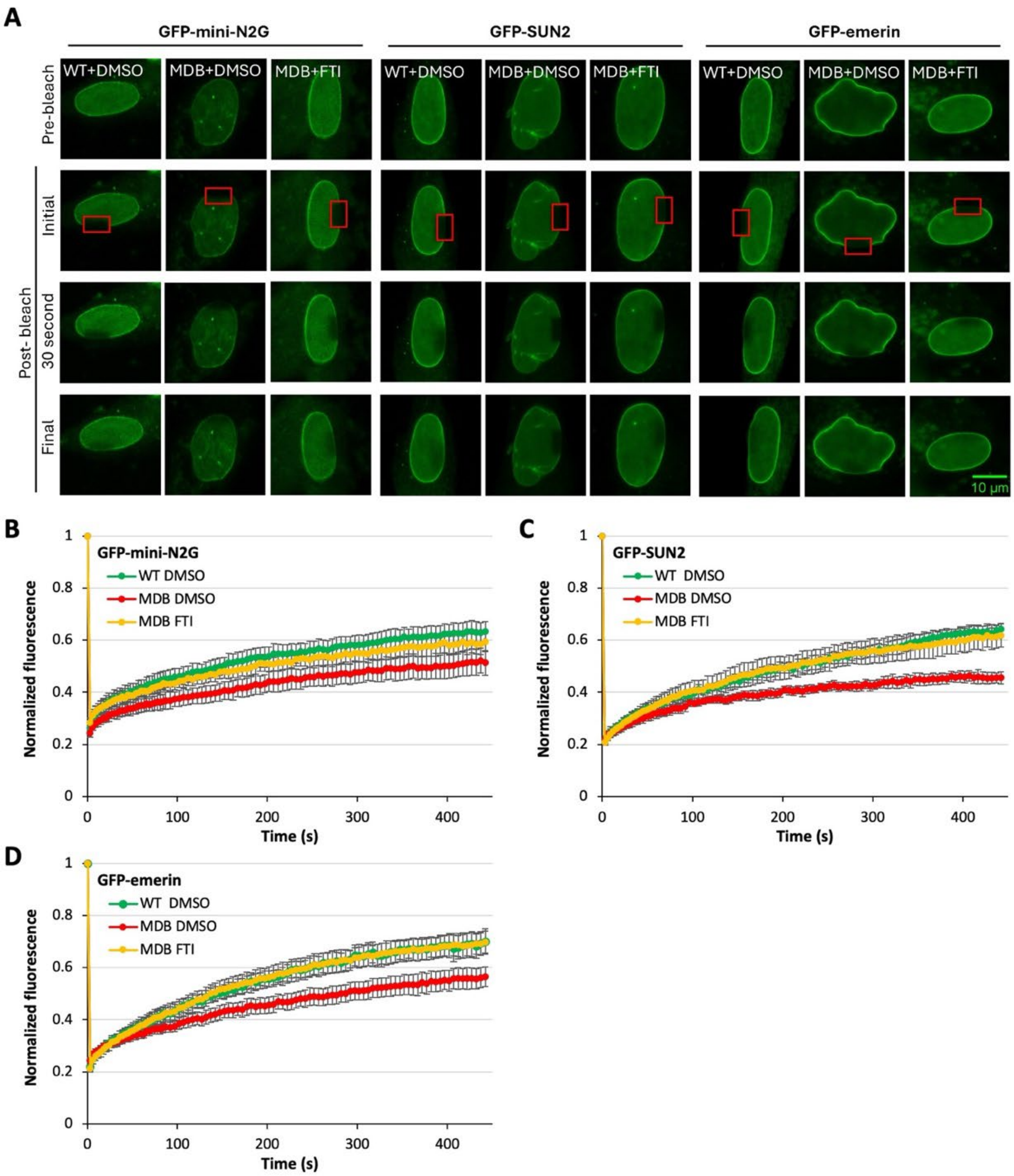

Fig. S3. Diffusional mobilities of mini-N2G, SUN2 and emerin in the nuclear envelope of MDB fibroblasts. (A) Representative confocal fluorescence images of WT and MDB fibroblasts expressing EGFP-tagged mini-N2G, SUN2 or emerin treated with FTI or DMSO (control) and subjected to FRAP. Images represent nuclear GFP fluorescence pre-bleach and at different times post-bleach as indicated. Final is 442 seconds post photobleaching. (B-D) Normalized FRAP intensities of EGFP-tagged mini-N2G (B), SUN2 (C) and emerin (D) in the nuclear envelope of WT and MDB fibroblasts treated with DMSO (control) or FTI-277 as indicated. Values are means ± SEM from *N*= 3 biological replicates.

Journal of Cell Science • Supplementary information

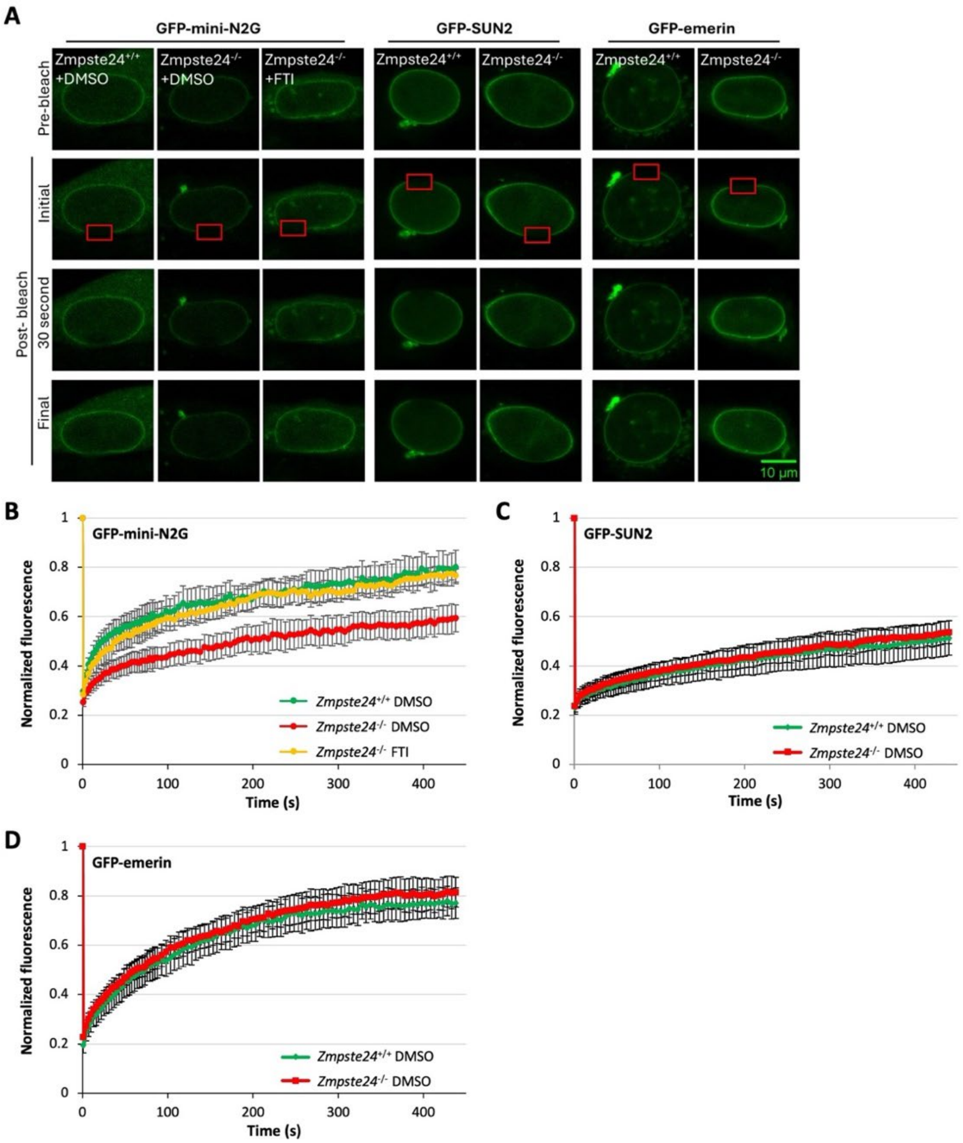

Fig. S4. Diffusional mobilities of mini-N2G, SUN2 and emerin in the nuclear envelope of *Zmpste24*$^{+/+}$ and *Zmpste24*$^{-/-}$ MEFs. (A) Representative confocal fluorescence images of *Zmpste24*$^{+/+}$ and *Zmpste24*$^{-/-}$ MEFs expressing EGFP-tagged mini-N2G, SUN2 or emerin subjected to FRAP. Cells expressing mini-N2G were treated with FTI or DMSO (control) but not cells expressing EGFP-SUN2 or EGFP-emerin as there were no differences in the diffusional mobilities in *Zmpste24*$^{+/+}$ and *Zmpste24*$^{-/-}$ MEFs. Images represent nuclear GFP fluorescence pre-bleach and at different times post-bleach as indicated. Final is 442 seconds post photobleaching. (B-D) Normalized FRAP intensities of EGFP-tagged mini-N2G (B), SUN2 (C) and emerin (D) in the nuclear envelope of *Zmpste24*$^{+/+}$ and *Zmpste24*$^{-/-}$ MEFs treated with DMSO (control) or FTI-277 as indicated. Values are means ± SEM from N = 3 biological replicates.

Journal of Cell Science • Supplementary information

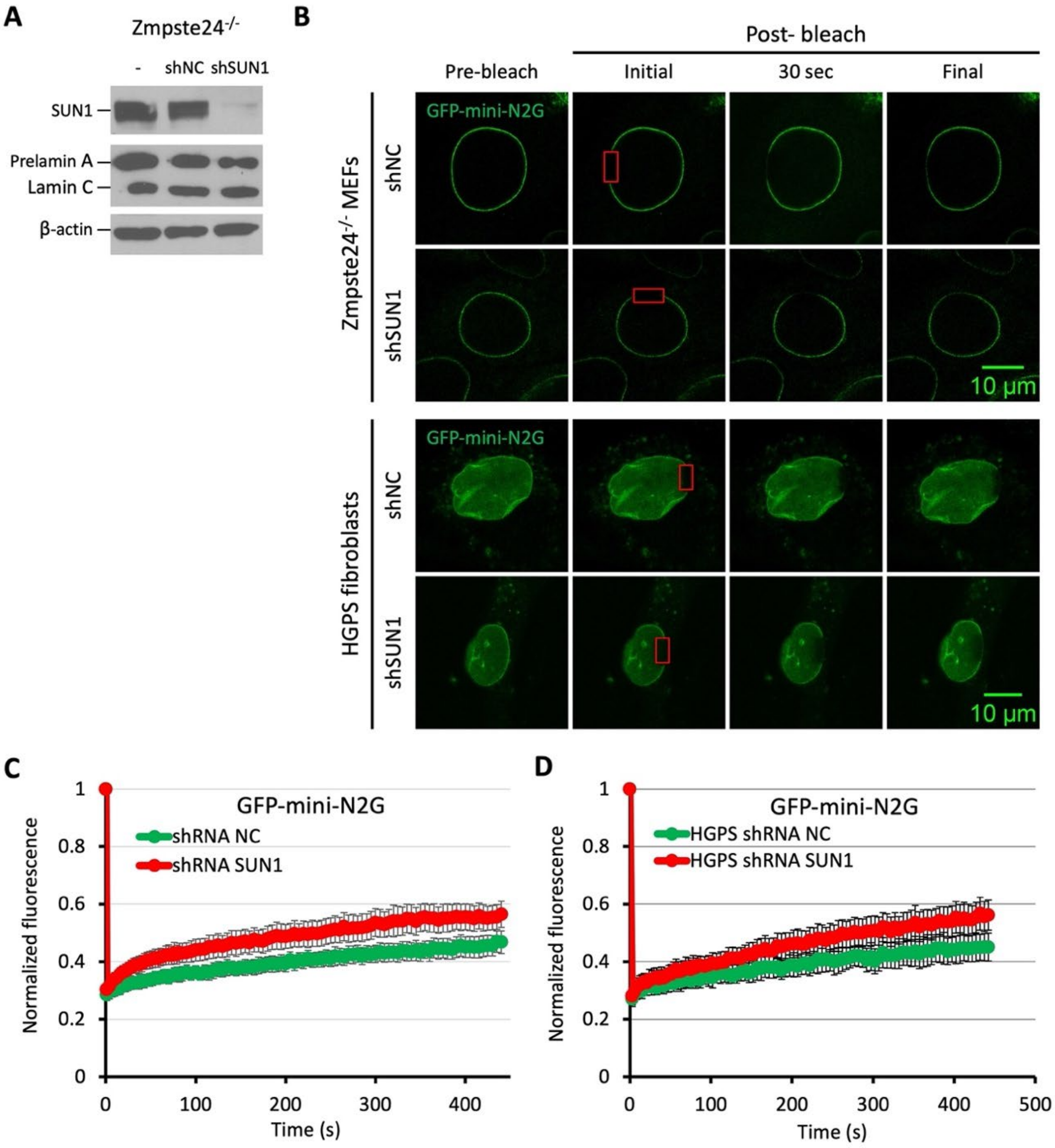

Fig. S5. Depletion of SUN1 in Zmpste24$^{-/-}$ MEFs or HGPS fibroblasts enhances diffusional mobilities of mini-N2G. (A) Immunoblot showing the depletion of SUN1 protein by SUN1 shRNA treatment (shSUN1) but not non-coding control (shNC). Blots were probed with the indicated antibodies on the left. (B) Representative confocal fluorescence images of Zmpste24$^{-/-}$ MEFs and HGPS fibroblasts expressing EGFP-tagged mini-N2G and the indicated shRNA showing FRAP at the indicated times pre- and post-bleach. (C, D) Normalized FRAP intensities of EGFP-mini-N2G in Zmpste24$^{-/-}$ MEFs (C) and HGPS fibroblasts (D) treated as indicated. Values are means ± SEM from $N = 3$ biological replicates.

Journal of Cell Science • Supplementary information

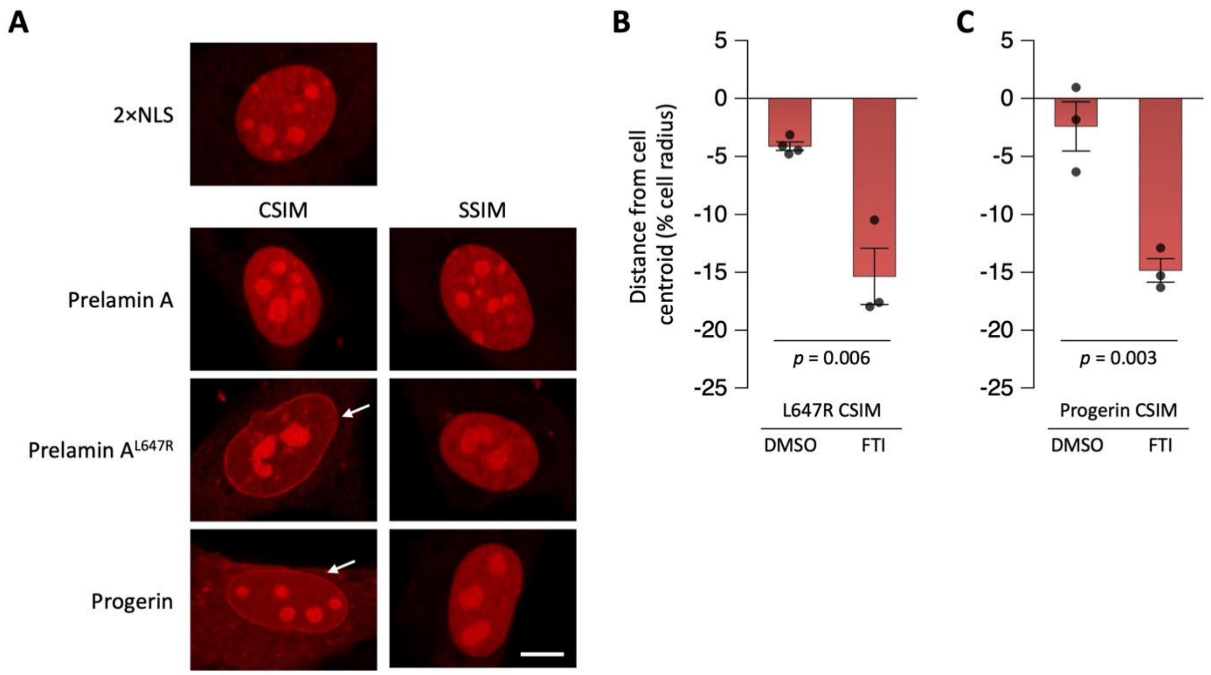

Fig. S6. Localization of the carboxyl-terminal tails of prelamin A and variants and their effect on nuclear position after FTI treatment. (A) Representative fluorescence images of cells expressing 2×NLS-mScarlet-tagged prelamin A tail constructs. The arrow highlights mScarlet signal at the nuclear rim. Scale bar: 10 µm. (B, C) Nuclear positions in LPA-stimulated NIH3T3 fibroblasts expressing the tail fragment of prelamin A L647R (B) and that of progerin (C) in the absence (DMSO) and presence of 2.5 µM FTI-277 (FTI). Values are means ± SEM from $N = 3$ biological replicates ($n > 90$ cells). p-values were calculated by 1-way ANOVA with Tukey's test.

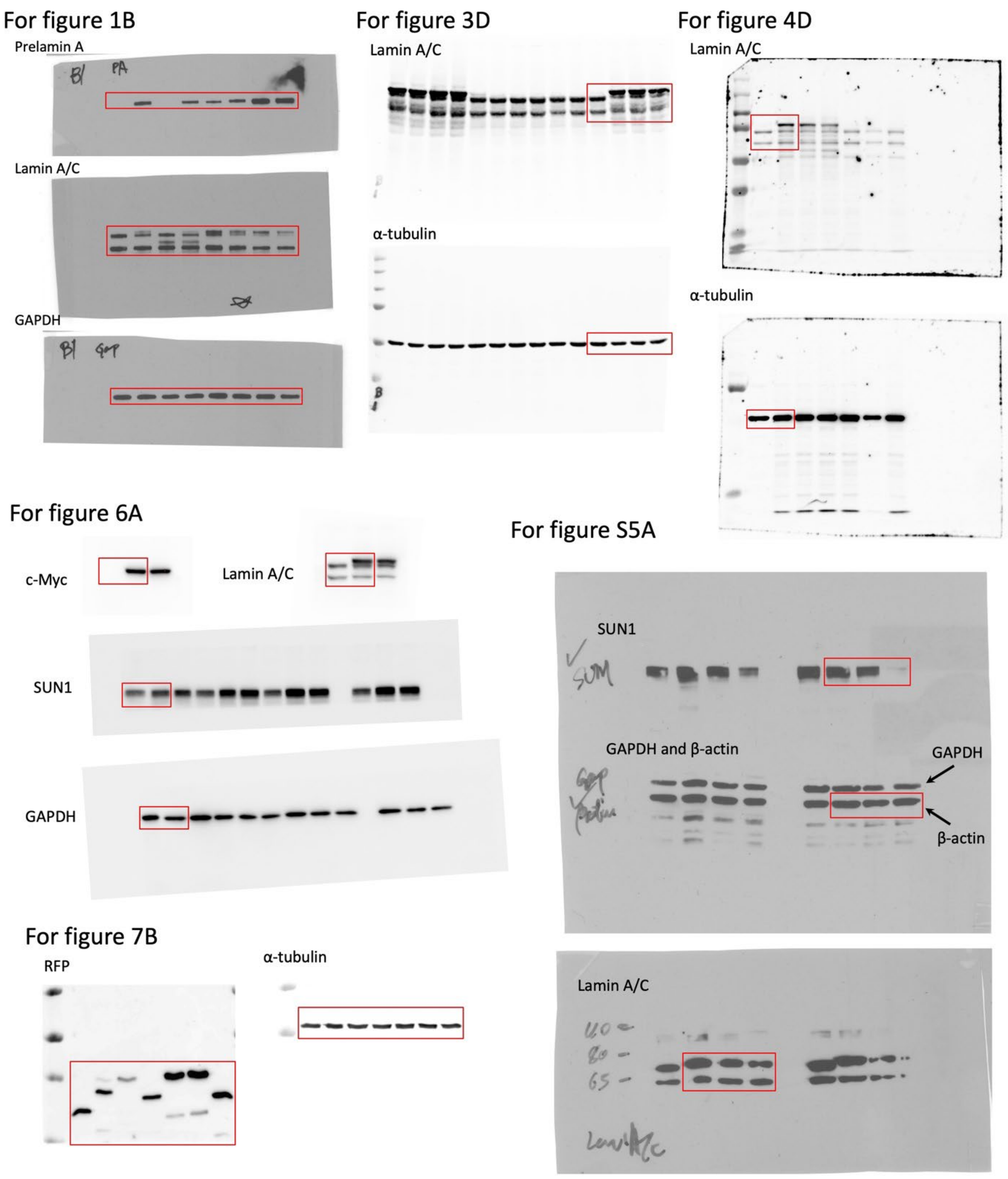

Fig. S7. Whole immunoblot results.

Journal of Cell Science • Supplementary information
